# Stress Hyperglycemia Drives CD4^+^ T Cell PANoptosis and Postoperative Organ Injury via Monocyte‐Derived Succinate

**DOI:** 10.1002/advs.76968

**Published:** 2026-08-05

**Authors:** Shuai Zhao, Xuemei Miao, Wei Luo, Tao Liu, Yang Wang, Jiaying Li, Di Fu, Weiyun Shen, Yan Luo, Kang Chen, Ruping Dai, Hui Li

**Affiliations:** ^1^ Department of Anesthesiology the Second Xiangya Hospital Central South University Changsha China; ^2^ Anesthesiology Research Institute of Central South University Changsha Changsha China; ^3^ Department of Anesthesiology XiangYa Hospital Central South University Changsha China; ^4^ Department of Obstetrics and Gynecology Wayne State University Detroit USA

**Keywords:** aortic dissection, CD4^+^ T cells, mitochondria, PANoptosis, succinate

## Abstract

Perioperative stress hyperglycemia is a transient but frequent metabolic disturbance strongly linked to postoperative organ injury and mortality; however, the immunometabolic mechanisms driving this association remain largely undefined. In a two‐center cohort of patients undergoing total aortic arch replacement, we identify stress hyperglycemia as an independent determinant of poor postoperative outcomes that associates strongly with CD4^+^ T cell loss. Hyperglycemia induces inflammatory PANoptosis in CD4^+^ T cells from patients in response to surgical trauma. This results from elevated glucose driving the accumulation and release of succinate from monocytes, which subsequently acts on CD4^+^ T cells to compromise mitochondrial integrity and activate ZBP1‐mediated PANoptosis. Our findings define a monocyte–T cell metabolic signaling axis that transduces hyperglycemic stress via elevated succinate to adaptive immune cell death and reveal potential therapeutic targets to prevent postoperative immune dysfunction and organ injury, especially for patients with hyperglycemic comorbidities.

## Graphical abstract

High glucose is linked to reduced succinate dehydrogenase activity in CD14^+^ monocytes, accompanied by succinate accumulation and extracellular release. Extracellular succinate exacerbates mitochondrial ROS production and mtDNA release in CD4^+^ T cells. Cytosolic mtDNA then activates Z‐DNA binding protein 1 (ZBP1) and engages ZBP1‐associated inflammatory PANoptosis. This process could disturb CD4^+^ T‐cell homeostasis and contribute to postoperative lymphopenia and postoperative organ injury.

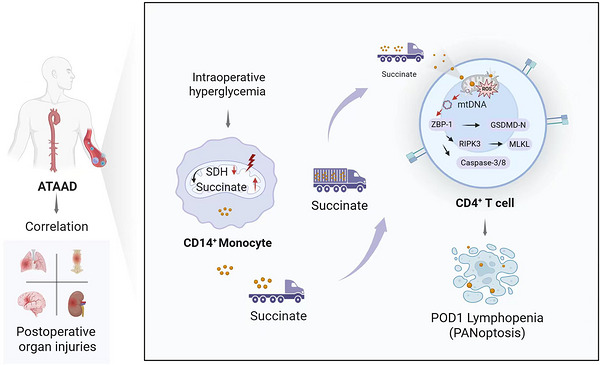

## 1 Introduction

Perioperative hyperglycemia is a common metabolic complication, affecting 20–40% of surgical patients [1, 2]. Even transient elevations in blood glucose are strongly associated with adverse outcomes, including increased mortality, prolonged ICU stay, and impaired recovery [3, 4, 5]. Notably, up to 30% of individuals without preexisting diabetes develop perioperative stress hyperglycemia. Compared to patients with established diabetes, those with stress hyperglycemia experience disproportionately worse outcomes, such as higher in‐hospital mortality and increased risk of poor functional recovery following hemorrhagic stroke [3, 4, 5, 6]. However, the mechanisms by which stress hyperglycemia drives adverse postoperative outcomes remain incompletely understood.

Immune dysregulation is increasingly recognized as a key determinant of organ injury after surgery [7]. Sustained or excessive hyperglycemia exerts broad immunosuppressive effects, compromising nearly all aspects of innate immunity and predisposing patients to infection [8, 9]. In patients with Stanford type‐A acute aortic dissection (ATAAD), a catastrophic condition with an in‐hospital mortality rate of 17–26% despite total aortic arch replacement surgery [10, 11], we recently demonstrated that hyperglycemia activates proinflammatory monocytes and contributes to poor recovery after surgery [12]. However, the impact of stress hyperglycemia on postoperative adaptive immunity remains largely undefined. Recent evidence, including both our work and that of others, has consistently identified CD4^+^ T cell lymphopenia as an independent risk factor for adverse prognosis in surgical and critical illness contexts [13, 14]. These observations warrant the key question of whether stress hyperglycemia disrupts CD4^+^ T cell homeostasis and impairs their function, thereby contributing to adverse postoperative outcomes.

While apoptosis is proposed as a major mechanism underlying perioperative CD4^+^ T cell lymphopenia [15], this condition develops rapidly in response to intense surgical trauma and often results in profound decline of CD4^+^ T cells together with increased circulating secreted and cellular biomarkers [16], which points to a coordinated multimodal cell death program that is reminiscent of PANoptosis, a newly recognized form of inflammatory programmed cell death mostly described in innate immune cells [17, 18]. PANoptosis is mediated by assembly of the PANoptosome complex that integrates pyroptosis, apoptosis, and necroptosis pathways and is orchestrated by Caspases and RIP kinases [19, 20, 21]. It has been implicated as an essential mechanism of innate immune defense and tissue injury during infections, inflammatory disorders, and cancer [19, 22]. Whether PANoptosis occurs in adaptive immune cells, particularly T cells, remains unknown.

Here, in a two‐center clinical cohort of ATAAD patients after total aortic arch replacement surgery, we find that stress hyperglycemia is an independent risk factor of adverse postoperative outcomes and strongly correlates with lymphopenia, with CD4^+^ T cells having the most profound decline in these patients. Hyperglycemia causes the accumulation and release of the tricarboxylic acid (TCA) cycle intermediate succinate from monocytes which damages the mitochondria of CD4^+^ T cells and activates the mitochondrial DNA (mtDNA)–cytosolic ZBP1 signaling cascade in CD4^+^ T cells to trigger PANoptosis. PANoptosis of CD4^+^ T cells grown in hyperglycemic conditions with monocytes was rescued by a combination of inhibitors to multiple cell death pathways or the reactive oxygen species (ROS) scavenger glutathione precursor N‐acetyl‐L‐cysteine (NAC). Our findings redefine the cellular landscape of PANoptosis and uncover a monocyte–T cell metabolic crosstalk that links stress‐induced hyperglycemia to adaptive immune cell death, revealing therapeutic targets to prevent postoperative immune dysfunction and organ injury.

## 2 Results

### 2.1 Prolonged Intraoperative Hyperglycemia Predicts Postoperative Lymphopenia and Organ Injury

**FIGURE 1:**
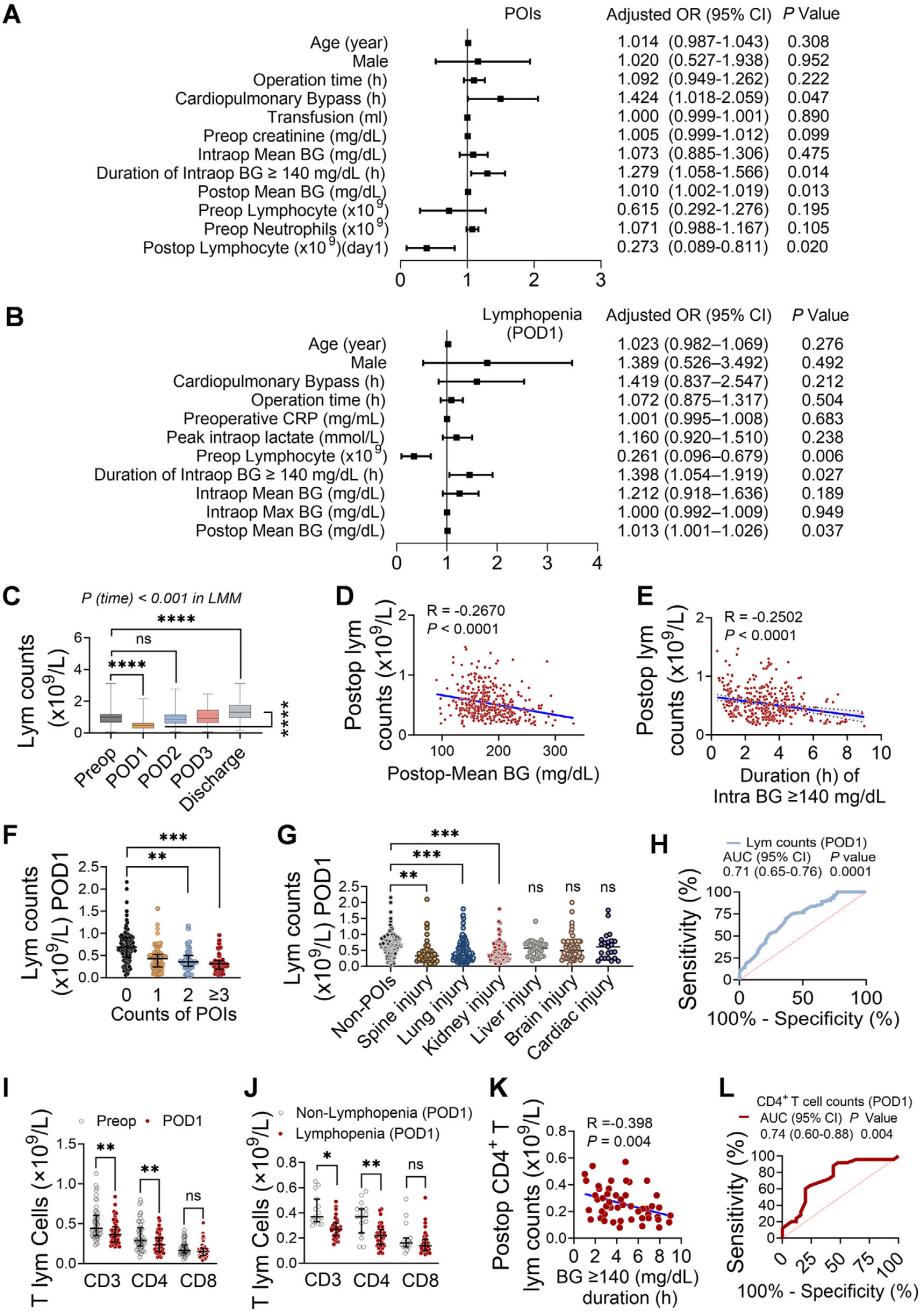
Association of intraoperative hyperglycemia with postoperative CD4^+^ T‐cell loss and organ injury in ATAAD patients. A, B) Multivariable logistic regression analyses identifying independent risk factors for postoperative organ injuries (POIs) (A) and postoperative day 1 (POD1) lymphopenia (B). Variables with *p* < 0.20 in univariable analyses were entered into the multivariable models. C) Longitudinal changes in circulating lymphocyte counts from the preoperative period (Preop) to discharge. D, E) Correlations of POD1 lymphocyte counts with the postoperative (Postop) mean glucose levels (D) and the duration of intraoperative blood glucose (BG) ≥140 mg/dL (E). F) Comparison of POD1 lymphocyte counts across patients grouped by the number of POIs. G) Comparison of POD1 lymphocyte counts among patients with specific organ injuries and those without any POIs. H) ROC analysis evaluating the predictive value of POD1 lymphocyte counts for POIs. I) Flow cytometric analysis of CD3^+^, CD4^+^, and CD8^+^ T cells before surgery and on POD1 (*n* = 50). J) Comparison of T‐cell subsets between patients with or without POD1 lymphopenia (*n* = 15/35, respectively). K) Correlation analysis between intraoperative (Intra) hyperglycemia duration and POD1 CD4^+^ T‐cell counts (*n* = 50). L) ROC analysis assessing the predictive performance of POD1 CD4^+^ T cell counts for POIs. Data are presented as median (IQR) or mean ± SEM. Statistical analyses were performed using a linear mixed‐effects model (C), Pearson correlation (D, E, K), one‐way ANOVA with Tukey's post hoc test (C, F, G), two‐tailed Student's *t*‐tests (I, J), and ROC analysis with AUC values (DeLong 95% CI; H, L). *p < 0.05, **p < 0.01, ***p < 0.001, ****p < 0.0001.

We analyzed the clinical profiles of 370 adults diagnosed with ATAAD (Table S1) who underwent total aortic arch replacement at two medical institutions (Figure S1). Postoperative organ injuries (POIs) occurred in 201 patients, accounting for 54.3% of the cohort (Table S1). The selection of variables was based on previous studies and restricted by data collection in the study [12, 15, 23, 24, 25]. Compared with patients without POIs, those who developed POIs exhibited a significantly longer duration of hyperglycemia (blood glucose, BG ≥ 140 mg/dL) during and after surgery (Table S1) [26]. Multivariate logistic regression identified four independent predictors of POIs, including prolonged intraoperative hyperglycemia, elevated postoperative mean glucose level, and lymphopenia on postoperative day 1 (POD1) and duration of cardiopulmonary bypass (Figure 1A and Table S2).

Given these associations, we examined whether perioperative stress hyperglycemia was linked to postoperative immune perturbation. Multivariate analysis revealed that both the duration of intraoperative hyperglycemia and the postoperative mean glucose level independently predicted lymphopenia on POD1 (Figure 1B and Tables S3 and S4), which corresponds to the perioperative nadir of lymphocyte counts (Figure 1C). Consistently, intraoperative and postoperative glucose levels positively correlated with each other (Figure S2A), and both showed significant negative correlations with total T cell counts on POD1 (Figure 1D,E). Although preoperative CRP differed between patients with and without POD1 lymphopenia, it was not independently associated with POD1 lymphopenia after multivariable adjustment (Table S3). Stratification by the number of affected organs revealed a stepwise association, with patients experiencing three or more POIs showing the lowest lymphocyte counts (Figure 1F). Further analysis of organ‐specific outcomes revealed that patients who developed spinal cord, lung or kidney injuries had significantly lower lymphocyte counts on POD1 (Figure 1G). Receiver operating characteristic (ROC) analysis indicated that lymphocyte counts on POD1 were significant predictors of POIs (AUC = 0.71, 95% CI: 0.65–0.76, p = 0.0001; Figure 1H).

To identify which lymphocyte subset primarily accounted for this postoperative decline and POIs, we performed flow cytometric analysis of peripheral blood collected before surgery and on POD1. Among the major T cell subsets, CD4^+^ T cells exhibited the pronounced reduction (Figure 1I). The CD4^+^ T cell loss was specific to patients with lymphopenia and was not seen in CD8^+^ T cells (Figure 1J). Notably, prolonged intraoperative hyperglycemia exceeding 140 mg/dL was strongly and inversely correlated with CD4^+^ T cell counts, but not with CD8^+^ T cell counts (Figure 1K and Figure S2B), suggesting that hyperglycemia may drive the selective loss of this lymphocyte subset. Consistently, decreased CD4^+^ T cell counts were strongly associated with POIs (AUC = 0.74; Figure 1L), but not with CD8^+^ T cell counts (AUC = 0.59, Figure S2C). Collectively, these findings show that early postoperative CD4^+^ T lymphopenia is closely linked to the development of organ injury in ATAAD patients and suggest that hyperglycemia‐associated immune dysregulation may contribute to postoperative immune complications involving early CD4^+^ T cell depletion.

### 2.2 Stress Hyperglycemia Correlates With Mitochondrial Dysfunction and Inflammatory PANoptosis in CD4^+^ T Cells

**FIGURE 2:**
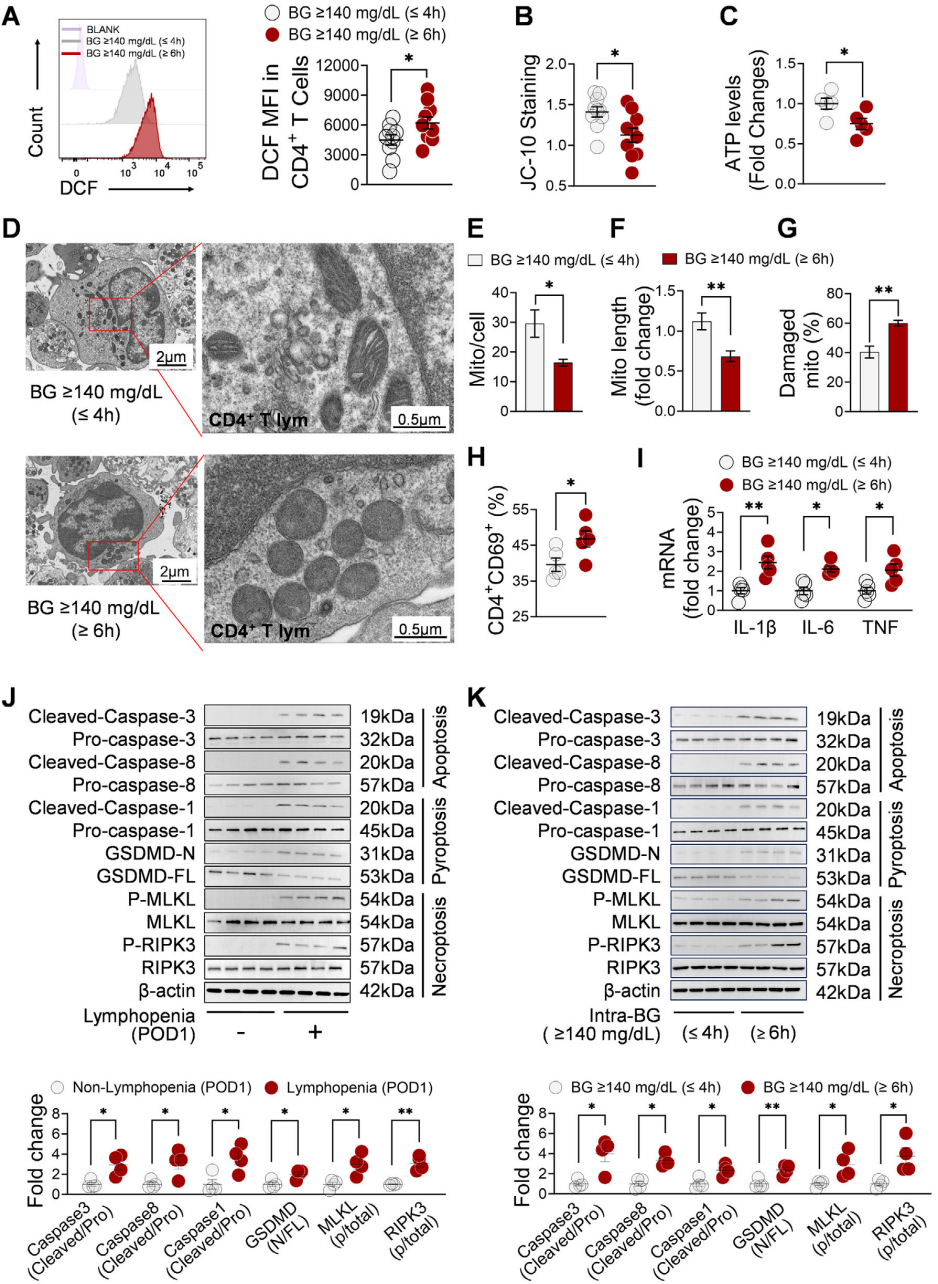
Postoperative CD4^+^ T cells from patients with stress hyperglycemia display mitochondrial dysfunction and inflammatory PANoptosis. A, B) Flow cytometric analysis of CD4^+^ T cell mitochondrial ROS (A) and mitochondrial membrane potential (B) in PBMCs from patients with varied durations of intraoperative hyperglycemia (n = 10 per group). C) Measurement of intracellular ATP levels in magnetically isolated CD4^+^ T cells from patients with varied durations of intraoperative hyperglycemia (n = 5). D‐G) Representative electron microscopy images (D) and quantification of mitochondrial (Mito) number (E), mitochondrial length (F) and the damaged mitochondria as indicated by the ultrastructural abnormalities (G) in isolated CD4^+^ T cells from patients with different durations of intraoperative blood glucose (BG) ≥140 mg/dL (n = 5 per group). H, I) Flow cytometry of CD69^+^ CD4^+^ T cell proportions in PBMCs (H) and RT–qPCR analysis of IL‐1β, IL‐6, and TNF mRNA expression in magnetically isolated CD4^+^ T cells from PBMCs (I) from patients stratified by intraoperative hyperglycemia duration (n = 5 per group). J) Western blot analysis and quantification of PANoptosis‐related protein expression in POD1 CD4^+^ T cells from patients with or without lymphopenia (n = 4 per group). K) Western blot analysis and quantification of the PANoptosis‐related protein in POD1 CD4^+^ T cells from patients with varied durations of intraoperative hyperglycemia (n = 4 per group). Data are shown as mean ± SEM. Statistical significance was determined using Student's *t*‐test. *p < 0.05, **p < 0.01.

Given the importance of mitochondrial function and integrity in T cell survival and activation [27, 28], we hypothesized that hyperglycemia‐induced metabolic stress may impair mitochondrial homeostasis to trigger cell death. CD4^+^ T cells from patients with prolonged intraoperative hyperglycemia exhibited increased mitochondrial reactive oxygen species (ROS) (Figure 2A), decreased mitochondrial membrane potential (Figure 2B), and reduced ATP production (Figure 2C). Transmission electron microscopy confirmed ultrastructural defects (Figure 2D), including reduced mitochondrial number (Figure 2E), shortened mitochondrial length (Figure 2F), and a higher proportion of damaged mitochondria as indicated by loss of cristae and/or ruptured inner or outer membranes (Figure 2G). The results suggest that sustained hyperglycemia is associated with profound mitochondrial impairment in CD4^+^ T cells.

Mitochondrial impairment in CD4^+^ T cells was accompanied by a pro‐inflammatory shift. In patients with sustained hyperglycemia, CD4^+^ T cells exhibited increased CD69 and CD25 expression, indicating enhanced T‐cell activation (Figure 2H and Figure S3), and elevated mRNA levels of interleukin‐1β (IL‐1β), IL‐6, and tumor necrosis factor (TNF) (Figure 2I). The levels of the cytokine were also higher in the blood of patients with POIs (Figure S4), linking metabolic stress to a pro‐inflammatory CD4^+^ T cell phenotype.

Given the concurrent mitochondrial dysfunction and pro‐inflammatory phenotype in CD4^+^ T cells, we then assessed whether the patients’ metabolic perturbations were associated with aberrant activation of cell death pathways. CD4^+^ T cells from lymphopenic patients exhibited increased levels of cleaved Caspase‐1 and Caspase‐3/8, Gasdermin D N‐terminal fragment (GSDMD‐N), phosphorylated MLKL, and RIPK3 (Figure 2J), indicating concurrent activation of pyroptotic, apoptotic, and necroptotic effectors consistent with PANoptosis. Patients with prolonged intraoperative hyperglycemia (≥6 h) showed higher levels of these PANoptosis‐associated proteins than those with shorter durations (≤4 h) (Figure 2K). These results strongly suggest that stress hyperglycemia may induce mitochondrial dysfunction, inflammatory activation, and PANoptosis in CD4^+^ T cells, possibly providing a mechanistic explanation for the observed lymphopenia and heightened inflammatory milieu associated with POIs in ATAAD patients.

### 2.3 High Glucose Induces Mitochondrial Dysfunction and PANoptosis in CD4^+^ T Cells in Vitro

**FIGURE 3:**
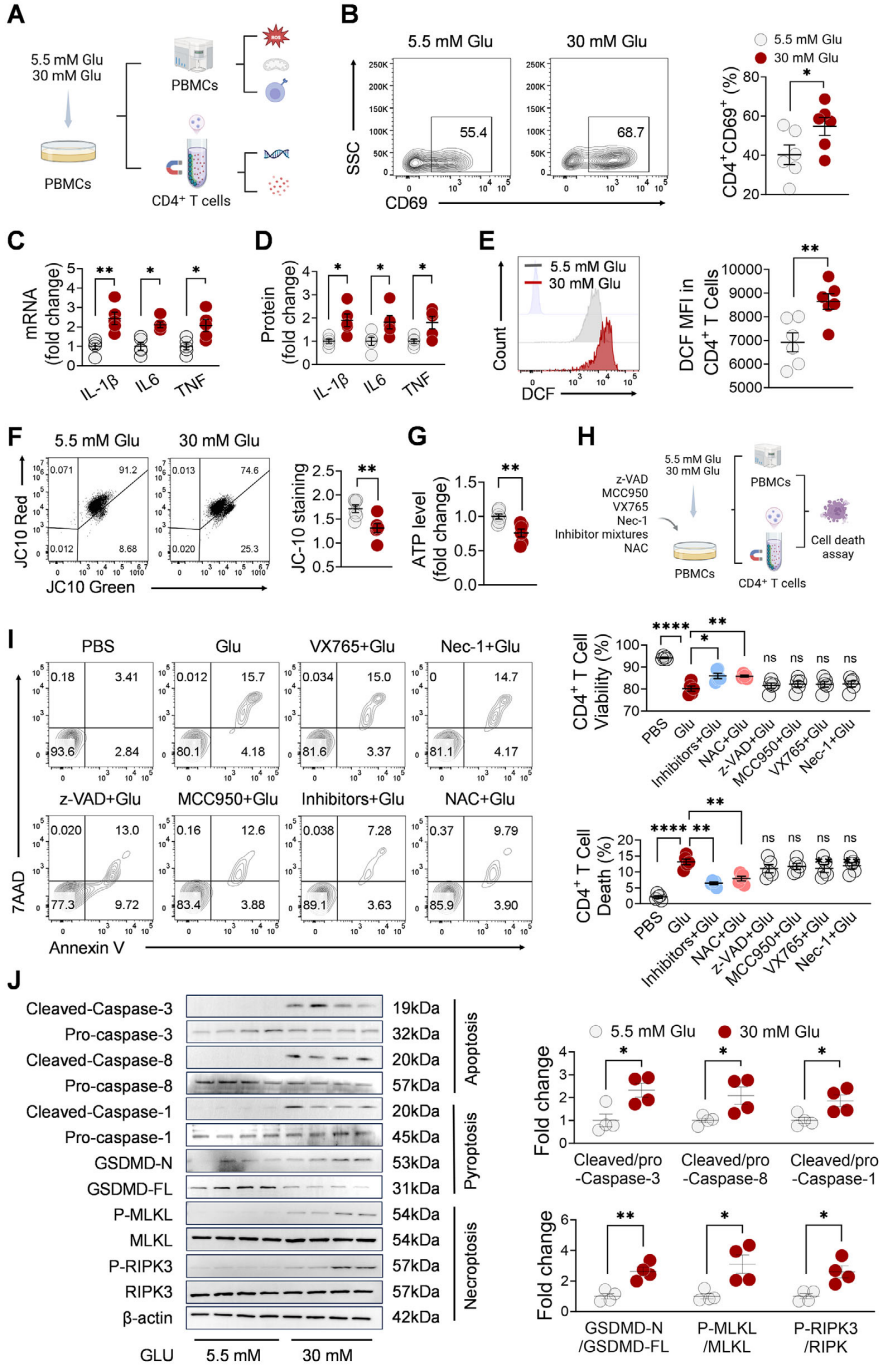
High glucose induces mitochondrial dysfunction and inflammatory PANoptosis in postoperative CD4^+^ T lymphocytes. A) Schematic of the experimental protocol for analyzing high glucose‐induced effects on CD4^+^ T cells. Peripheral blood mononuclear cells (PBMCs) were treated with 30 mM glucose for 24 h, after which CD4^+^ T cells were analyzed by flow cytometry (B, E, F) or sorted for downstream assays (C, D, G). B) Flow cytometric analysis of CD69^+^ CD4^+^ T cell proportions after in vitro stimulation with 5.5 mM or 30 mM glucose (n = 6 per group). C, D) RT–qPCR (C) and ELISA (D) quantification of IL‐1β, IL‐6, and TNF expression in CD4^+^ T cells following high‐glucose stimulation (n = 5 per group). E‐G) Flow cytometric assessment of mitochondrial ROS (E), mitochondrial membrane potential (F), and measurement of intracellular ATP levels (G) in CD4^+^ T cells from patients after high‐glucose exposure (n = 6 per group). H) Schematic diagram of the experimental protocol for analyzing high glucose‐induced effects on CD4^+^ T cell death in cultured PBMCs from patients. I) Flow cytometric analysis of CD4^+^ T cell viability and death in PBMCs treated with high glucose for 24 h, with or without pharmacological inhibitors (zVAD, MCC950, VX‐765, Nec‐1, NAC, or a combination) (n = 5 per group). J) Western blot analysis of apoptosis‐ (pro/cleaved caspase‐3 and ‐8), pyroptosis‐ (pro/cleaved caspase‐1 and GSDMD‐FL/N), and necroptosis‐related proteins (phosphorylated/total MLKL and RIPK3) in CD4^+^ T cells that were isolated from PBMCs cultured with 5.5 mM or 30 mM glucose for 24 h (n = 4 per group). Data are presented as mean ± SEM. Statistical significance was determined using Student's *t*‐test (B to G, J) or One‐way ANOVA followed by Bioferroni Post‐hoc test (I). *p < 0.05, **p < 0.01, ****p < 0.0001.

To determine how hyperglycemia affects CD4^+^ T cells, we cultured PBMCs from ATAAD patients in high‐glucose conditions. Mitochondrial function was assessed directly in PBMCs by flow cytometry, whereas CD4^+^ T cells were subsequently isolated from these PBMCs cultures and analyzed for gene expression, protein abundance, and ATP levels (Figure 3A). CD4^+^ T cells in PBMCs cultured in 30 mM glucose exhibited increased CD69 and CD25 expression (Figure 3B and Figure S5) and elevated IL‐1β, IL‐6, and TNF mRNA, and protein levels (Figure 3C,D). High‐glucose exposure also induced mitochondrial ROS accumulation (Figure 3E), loss of membrane potential (Figure 3F), reduced ATP production (Figure 3G) and cell death (Figure 3H,I). These effects were not detected in cells cultured in an isotropic medium (Figure S6). We next investigate which pathways implicates into the CD4^+^ T cell death resulted from hyperglycemia. Notably, cell death was significantly inhibited only by the combination of z‐VAD (a pan‐Caspase inhibitor), MCC950 (a NLRP3 inflammasome inhibitor), VX765 (a Caspase‐1/4 inhibitor), and Nec‐1 (a RIPK1 inhibitor), or by the ROS scavenger NAC, whereas single‐pathway inhibition had no protective effect (Figure 3I). Consistently, we observed simultaneous activation of Caspase‐1/3/8, GSDMD, RIPK3, and MLKL in the CD4^+^ T cells isolated from high‐glucose‐treated PBMCs (Figure 3J). These data indicate that high glucose triggers ROS‐dependent PANoptosis rather than a single cell death pathway. To ascertain whether these effects were intrinsic to T cells or dependent on other immune populations, we purified patients’ CD4^+^ T cells and cultured them in high‐glucose medium. In contrast to cultured PBMCs, cultured pure CD4^+^ T cells did not undergo death in high‐glucose condition (Figure S7). This result indicates that high‐glucose‐induced PANoptosis requires additional cellular and molecular signals beyond CD4^+^ T cells alone.

### 2.4 Postoperative Succinate Correlates With Hyperglycemia and Drives CD4^+^ T Cell PANoptosis

**FIGURE 4:**
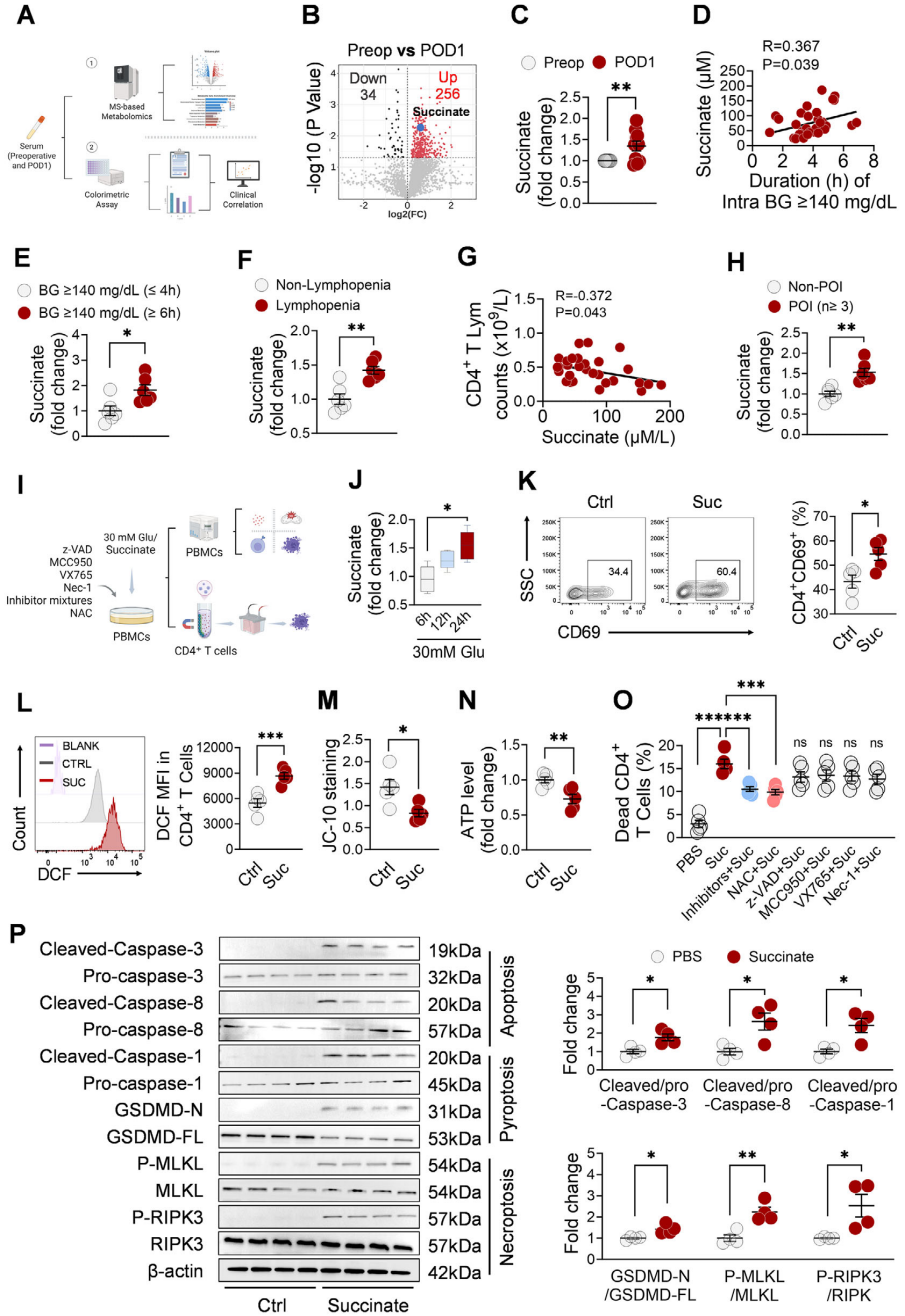
Succinate drives mitochondrial dysfunction and PANoptosis in CD4^+^ T cells. A) Schematic diagram of the study design. B, C) Metabolomic screening (B) and validation of serum succinate levels at baseline and POD1 (C) (n = 10 per group). D) Correlation between POD1 serum succinate levels and the duration of intraoperative hyperglycemia (blood glucose ≥140 mg/dL). E) Comparison of intracellular succinate level in CD4^+^ T cells from POD1 patients with intraoperative BG ≥140 mg/dL less than 4 h or more than 6 h (n = 6 per group). F) Comparison of serum succinate levels in POD1 patients with normal lymphocyte counts versus lymphopenia (n = 6 per group). G) Association between POD1 serum succinate levels and postoperative CD4^+^ T cell counts (n = 30). H) Comparison of POD1 serum succinate levels in patients with or without POI (n = 6 per group). I) Schematic diagram of the experimental protocol for succinate‐induced CD4^+^ T cell dysfunction and PANoptosis. J) Intracellular succinate in CD4^+^ T cells isolated after PBMC culture under 30 mM glucose for 6, 12, or 24 h (n = 5 per group). K) CD4^+^ T cells stimulated with PHA with or without succinate for 24 h and analyzed for activation (%CD69^+^) by flow cytometry (n = 5 per group). L‐N) mitochondrial ROS (L), mitochondrial membrane potential (M), and intracellular ATP levels (N) in CD4^+^ T cells treated with PBS or succinate (10 mM) for 24 h (n = 5 per group). O) Cell death in CD4^+^ T cells pretreated with cell death inhibitors or NAC followed by succinate stimulation (10 mM) (n = 5 per group). P) Western blot analysis of apoptosis (cleaved/pro–caspase‐3 and ‐8), pyroptosis (GSDMD‐N/GSDMD‐FL, cleaved/pro–caspase‐1), and necroptosis (p‐MLKL/MLKL, p‐RIPK3/RIPK3) in CD4^+^ T cells after 24 h of PBS or succinate (10 mM) treatment (n = 4 per group). Data are presented as mean ± SEM. Statistical significance was determined by one‐way ANOVA (O), Student's *t*‐test (C, E, F, H, K, L, M, N, P) and Pearson correlation (D, G). *P < 0.05, **P < 0.01, ***P < 0.001.

To identify the metabolic signals driving postoperative CD4^+^ T cell PANoptosis, we performed metabolomic analysis of circulating metabolites in postoperative patients (Figure 4A). A total of 290 circulating metabolites were significantly altered in postoperative patients compared to the baseline, with 256 upregulated and 34 downregulated (Figure 4B). Metabolite Set Enrichment Analysis (MSEA) revealed that the most significantly enriched metabolic pathways were primarily associated with Thiamine metabolism and, importantly, mitochondrial electron transporter chain (Figure S8), indicating a critical metabolic link for subsequent mechanistic investigation of CD4^+^ T cell dysfunction. Among these changes, succinate was one of the most profoundly upregulated metabolites. Given its critical role as a TCA cycle intermediate linking metabolism and immune signaling, we examined the succinate level perioperatively in ATAAD patients. Succinate levels in the sera on POD1 were markedly elevated compared with preoperative levels (Figure 4C) and correlated positively with the duration of intraoperative hyperglycemia (Figure 4D,E). Moreover, postoperative succinate concentrations were significantly higher in patients with lymphopenia (Figure 4F) and negatively correlated with circulating CD4^+^ T cell counts (Figure 4G). Patients who developed POIs exhibited higher POD1 succinate levels (Figure 4H), which also correlated with longer ICU stays (Figure S9), underscoring the elevated succinate as a critical metabolic signal linking postoperative lymphopenia and POIs.

We next cultured patient PBMCs in high glucose and subsequently purified CD4^+^ T cells to examine their intracellular succinate level (Figure 4I). Twenty‐four hours of high glucose treatment significantly increased intracellular succinate accumulation in the CD4^+^ T cells (Figure 4J). Treatment of CD4^+^ T cells with exogenous succinate induced an immunometabolic phenotype resembling that seen in CD4^+^ T cells of postoperative lymphopenic patients and high‐glucose‐treated PBMCs, including increased CD69 and CD25 expression (Figure 4I,K and Figure S10), mitochondrial ROS level (Figure 4L), mitochondria membrane depolarization (Figure 4M) and decreased intracellular ATP level (Figure 4N). These changes are associated with increased cell death, which was rescued by NAC or a combination of inhibitors including z‐VAD, MCC950, VX765 and Nec‐1, to multiple cell death pathways (Figure 4O). Succinate‐treated CD4^+^ T cells from ATAAD patients displayed hallmarks of PANoptosis, including activated Caspase‐1/3/8, cleaved GSDMD, and phosphorylated RIPK3 and MLKL (Figure 4P). Therefore, succinate acts as a metabolic mediator linking hyperglycemia to CD4^+^ T cell mitochondrial injury and inflammatory PANoptosis.

### 2.5 Succinate Induces mtDNA Release to Trigger CD4+ T Cell PANoptosis via ZBP1

**FIGURE 5:**
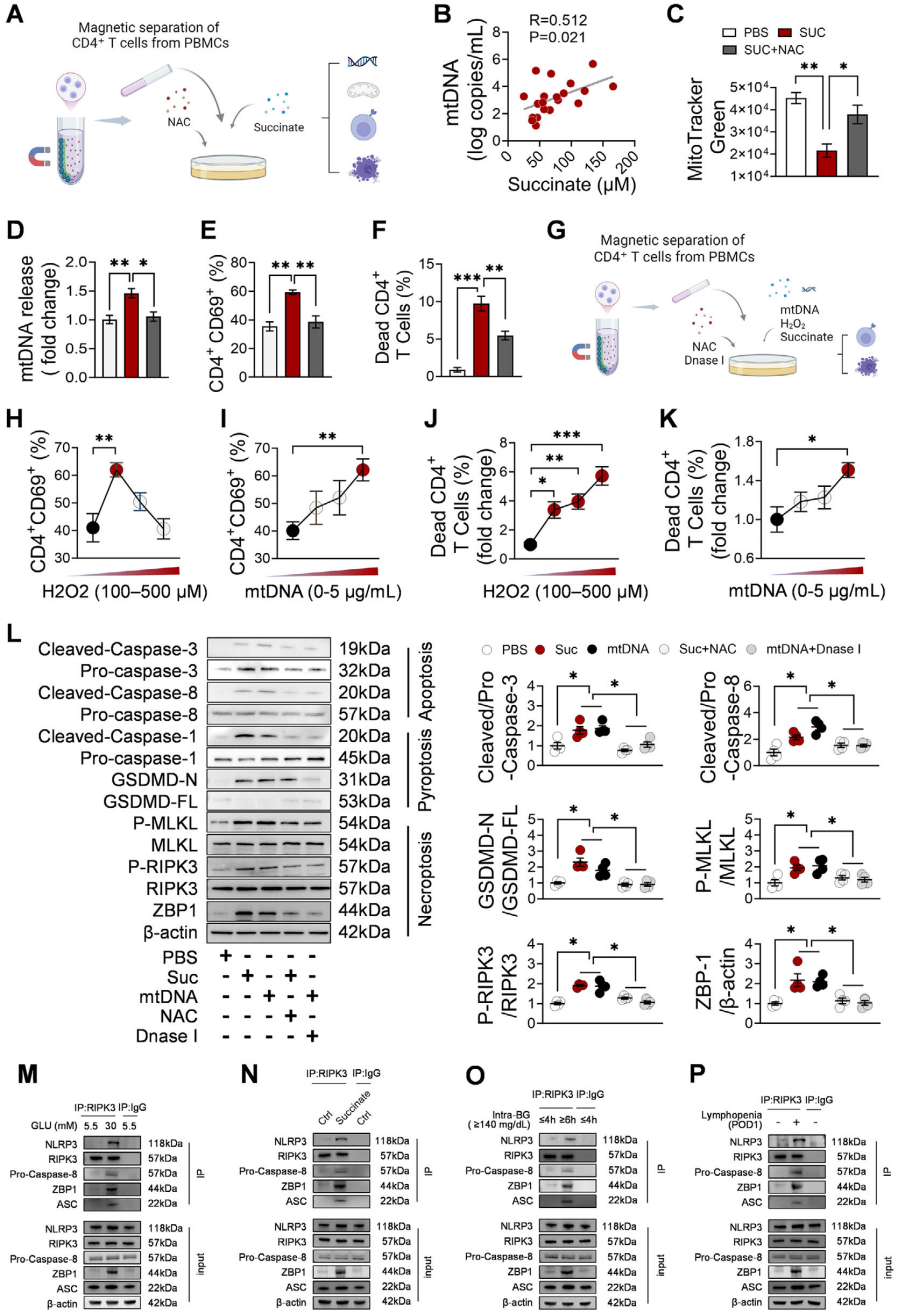
Succinate‐induced mtDNA release activates ZBP1‐dependent inflammatory PANoptosis in CD4^+^ T cells. A) Schematic diagram of the experimental protocol for succinate‐induced CD4^+^ T cell mitochondria dysfunctions in cultured CD4^+^ T cells. B) Correlation between serum succinate (SUC) and mtDNA levels on POD1 (n = 20). C‐F) CD4^+^ T cells treated with succinate (10 mM) in the presence or absence of NAC (20 mM) for 24 h (n = 6 per group) were analyzed for mitochondrial mass (C), extracellular mtDNA (D), activation (E), and cell death (F). G) Schematic diagram of the role of mtDNA in succinate ‐induced CD4^+^ T cell PANoptosis. H‐K) CD4^+^ T cells were exposed for 24 h to increasing doses of H_2_O_2_ (100, 300, 500 µM) or mtDNA (0, 1, 3, 5 µg/mL), and CD4^+^ T cell activation (H and I) and cell death (J and K) were quantified by flow cytometry (n = 5 per group). L) Western blot analysis of ZBP1, GSDMD‐N, p‐RIPK3, and p‐MLKL in CD4^+^ T cells stimulated for 24 h with succinate (10 mM) or mtDNA (5 µg/mL), alone or in combination with NAC (20 mM), or DNase I (100 U/mL) (n = 4 per group). M) Co‐immunoprecipitation analysis of PANoptosome‐associated proteins (NLRP3, RIPK3, pro‐caspase‐8, ZBP1, ASC) in CD4^+^ T cells sorted from PBMCs after culture with 5.5 mM or 30 mM glucose for 24 h. N) Co‐immunoprecipitation analysis of PANoptosome‐associated proteins in sorted CD4^+^ T cells stimulated with 10 mM succinate for 24 h. O, P) Co‐immunoprecipitation analysis of PANoptosome‐associated proteins in sorted POD1 CD4^+^ T cells from patients stratified by the duration of intraoperative blood glucose ≥140 mg/dL (O) or by postoperative lymphopenia on POD1 (P). Data are presented as mean ± SEM. Statistical significance was determined by Pearson correlation (B) and one‐way ANOVA (C to F, H to L). *P < 0.05, **P < 0.01, ***P < 0.001.

Given succinate‐induced mitochondrial damage in CD4^+^ T cells, we asked whether succinate promotes the release of mtDNA, which has emerged as a key trigger of PANoptosis, in CD4^+^ T cells (Figure 5A). Serum mtDNA levels on POD1 positively correlated with circulating succinate levels (Figure 5B). In line with this observation, succinate treatment of cultured CD4^+^ T cells reduced intracellular mitochondria mass and increased extracellular mtDNA, both of which were prevented by NAC (Figure 5C,D). In addition, succinate treatment heightened CD4^+^ T cell activation and incrased cell death, both of which were also reversed by the addition of NAC (Figure 5E,F). Exposure of CD4^+^ T cells to H_2_O_2_ or transfection of mtDNA into the cytosol resulted in increased CD69 expression (Figure 5G–I) and cell dealth (Figure 5J,K) similarly to succinate treatment. Both succinate and the transfectin of mtDNA upregulated the expression of ZBP1, a critical scaffold for nucleating the PANoptosome complex, in CD4^+^ T cells and caused the activation of its downstream effectors, including the cleavage of Caspase‐1/3/8 and GSDMD and the phosphorylation of RIPK3 and MLKL (Figure 5L). These events were abolished by NAC and DNase I treatment (Figure 5L). Consistently, ATAAD patients with prolonged intraoperative hyperglycemia or POIs had significantly elevated level of circulating mtDNA (Figure S11A,B) and increased ZBP1 protein in CD4^+^ T cells (Figure S11C,D).

To further assess the formation of a PANoptotic signaling complex, we performed co‐immunoprecipitation analysis in CD4^+^ T cells. Both high‐glucose and succinate stimulation enhanced the association of RIPK3 with ZBP1, ASC, pro‐caspase‐8, and NLRP3, consistent with the assembly of a PANoptotic signaling complex in CD4^+^ T cells (Figure 5M,N). Consistently, this complex was more evident in POD1 CD4^+^ T cells from patients with prolonged intraoperative hyperglycemia, particularly those with blood glucose ≥140 mg/dL for more than 6 h, and from patients who developed postoperative lymphopenia (Figure 5O,P). These data link the dysregulated succinate‐mtDNA‐ZBP1 axis to the adverse immune outcomes observed in response to surgical trauma.

### 2.6 Hyperglycemia Represses SDH Expression and Elevates Succinate in Monocytes

**FIGURE 6:**
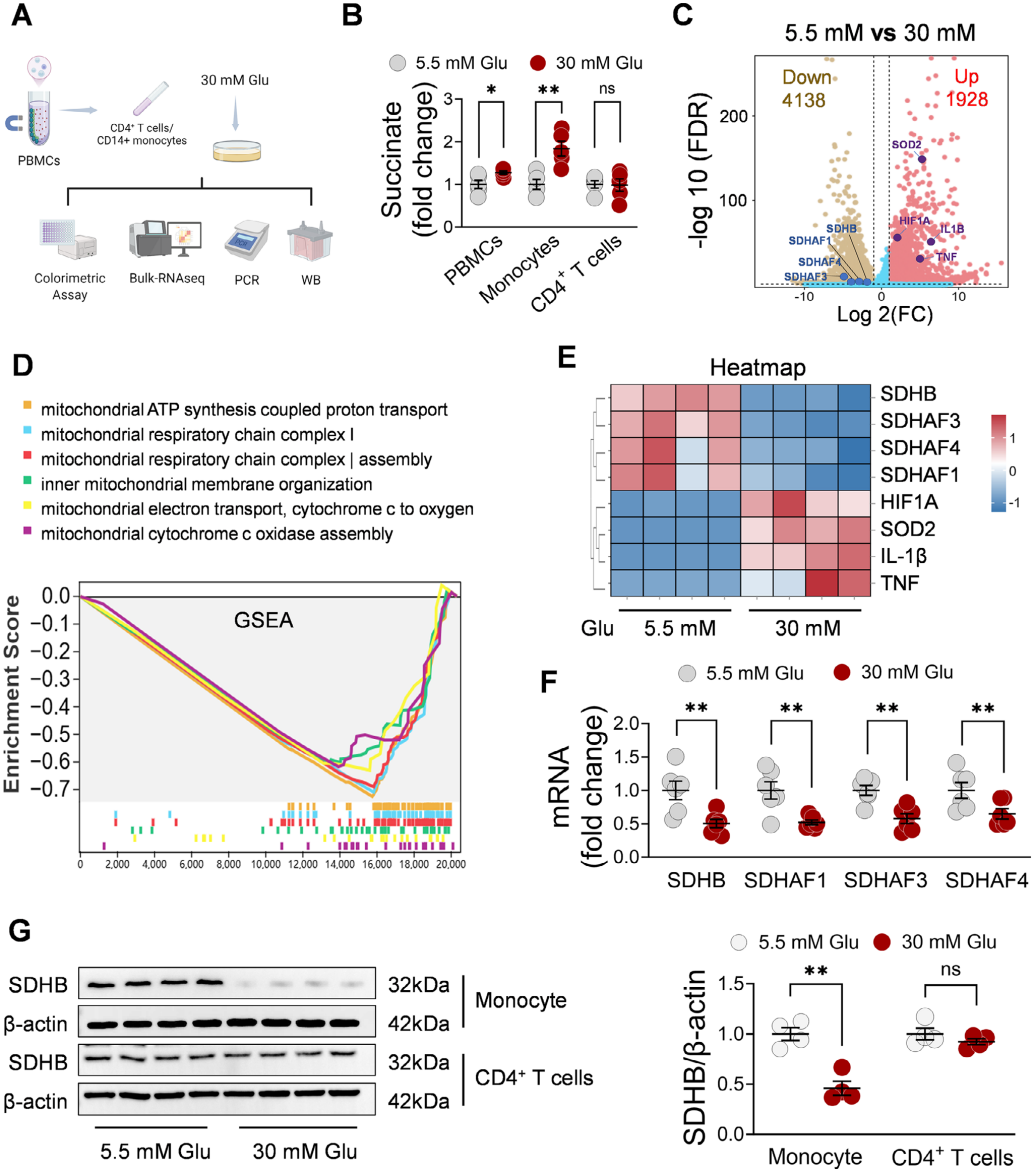
High glucose suppresses SDH expression and promotes succinate accumulation in postoperative CD14^+^ monocytes. A) Schematic diagram of the experimental protocol for cellular origin of succinate and its mechanism after high glucose exposure. B) Intracellular succinate levels in CD14^+^ monocytes, and CD4^+^ T cells from ATAAD patients after 24 h of culture in 5.5 mM or 30 mM glucose (Glu) (n = 5 per group). C‐E) RNA sequencing of CD14^+^ monocytes cultured in 5.5 mM or 30 mM glucose for 24 h (n = 4 per group), showing a volcano plot of differentially expressed genes (C), GSEA highlighting mitochondrial dysfunction signatures (D), and a heatmap of succinate‐associated genes (E). F) RT–qPCR analysis of SDH subunits genes in CD14^+^ monocytes following high‐glucose exposure (n = 6 group). G) Western blot analysis of SDH protein expression in CD14^+^ monocytes and CD4^+^ T cells after 24 h of culture in 5.5 mM or 30 mM glucose (n = 4 per group). Data are presented as mean ± SEM. Statistical significance was determined using two‐tailed Student's *t*‐tests. *p < 0.05, **p < 0.01.

To identify the cellular source of elevated succinate in response to surgical trauma, we first performed sorting of major immune cell populations from PBMCs of ATAAD patients, including CD4^+^ T cells and monocytes (Figure S12), and cultured them in normal or high glucose media (Figure 6A). Monocytes exhibited pronounced intracellular succinate accumulation, whereas CD4^+^ T cells showed minimal changes (Figure 6B), suggesting that monocytes may be an important source of hyperglycemia‐induced succinate. We then performed RNA sequencing (RNA‐Seq) of monocytes cultured in normal or high glucose and identified 1928 genes upregulated and 4138 genes downregulated by high‐glucose treatment (Figure 6C). Gene set enrichment analysis (GSEA) revealed enrichment of pathways related to mitochondrial inner membrane disruption and respiratory chain imbalance of high‐glucose‐treated monocytes (Figure 6D). High‐glucose downregulated the gene encoding the catalytic iron‐sulfur protein subunit (subunit B) of succinate dehydrogenase (*SDHB*), which converts succinate to fumarate in the TCA cycle, and the genes encoding factors for the assembly of the SDH enzyme, *SDHAF1*, *SDHAF3* and *SDHAF4*, while upregulated genes encoding superoxide dismutase (*SOD*), hypoxia‐inducible factor 1 alpha (*HIF1A*) and the inflammatory cytokines IL‐1β and TNF (*TNF*) (Figure 6E and Figure S13). Given that SDH is the main succinate‐metabolizing enzyme whose dysregulation is implicated in succinate accumulation, we focused on its gene and protein expression in response to high glucose treatment (Figure 6F,G). Consistent with our transcriptomic findings, SDH expression was specifically suppressed in monocytes but remained unchanged in CD4^+^ T cells (Figure 6G and Figure S14). Thus, hyperglycemia induces a metabolically reprogrammed monocyte state characterized by succinate accumulation and repressed SDH expression.

### 2.7 Monocyte Succinate Mediates High‐glucose‐induced PANoptosis of CD4^+^ T Cells

**FIGURE 7:**
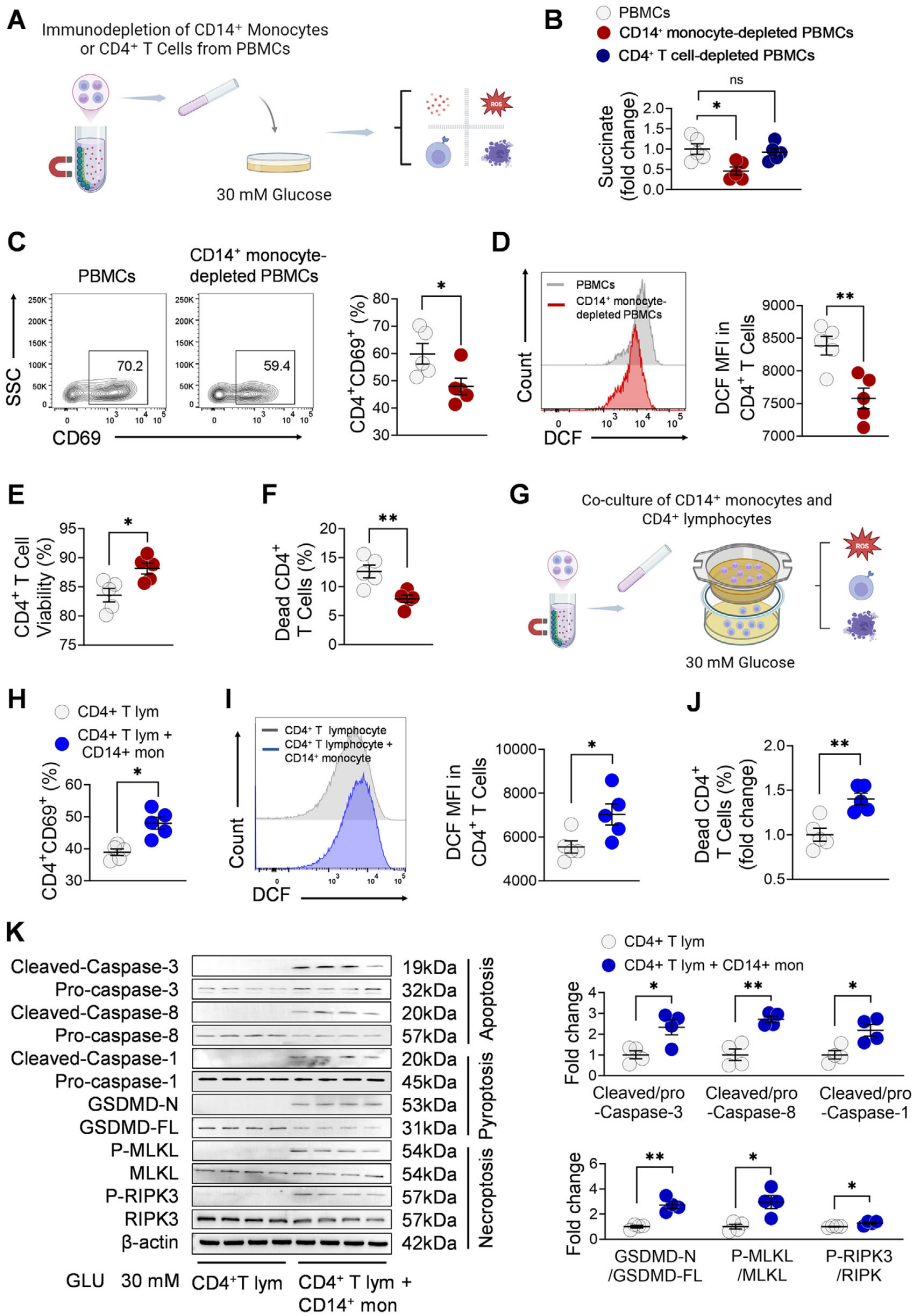
Monocyte‐derived succinate drives high‐glucose–induced mitochondrial dysfunction and PANoptosis in postoperative CD4^+^ T cells. A) Schematic diagram of the experimental protocol to evaluate the monocyte‐CD4^+^ T cell crosstalk mediating high glucose‐induced effects. B) Succinate levels in the culture supernatants were measured in PBMCs with different cell depletion under high‐glucose conditions (30 mM, 24 h; n = 5 per group). C‐F) CD4^+^ T‐cell activation (C), ROS production (D), viability (Annexin V^−^ 7‐AAD^−^; E) and cell death (Annexin V^+^ 7‐AAD^+^; F) were evaluated by flow cytometry (n = 5 per group). G) Schematic protocol for analyzing CD14^+^ monocyte‐CD4^+^ T cell co‐culture under high glucose conditions. H‐J) CD4^+^ T‐cell activation (H), ROS levels (I), and cell death (J) were quantified by flow cytometry (n = 5 per group). K) Western blot analysis of apoptotic (cleaved/pro–caspase‐3 and ‐8), pyroptotic (GSDMD‐N/GSDMD‐FL and cleaved/pro–caspase‐1), and necroptotic (p‐MLKL/MLKL and p‐RIPK3/RIPK3) markers in CD4^+^ T cells cultured under high‐glucose conditions alone or in co‐culture with CD14^+^ monocytes (n = 4 per group). Data are presented as mean ± SEM. Statistical significance was determined using one‐way ANOVA (B) or a two‐tailed Student's *t*‐test (C to F, H to K). *p < 0.05, **p < 0.01.

To further assess the functional contribution of monocyte‐derived succinate, we performed depletion of specific cell populations in PBMCs from ATAAD patients and examined its impact on high‐glucose‐induced succinate levels in the culture (Figure 7A). While the depletion of CD4^+^ T cells had no effect on succinate levels in the PBMC culture, depletion of monocytes completely abolished high‐glucose‐induced succinate accumulation in PBMC cultures (Figure 7B), with concomitantly reduced CD69 expression (Figure 7C), ROS level (Figure 7D) and death of CD4^+^ T cells (Figure 7E,F). These data show that monocytes are the major source of succinate in PBMC response to high glucose. We then cocultured monocytes with CD4^+^ T cells in high‐glucose medium (Figure 7G), CD4^+^ T cells cocultured with monocytes had increased level of CD69 and CD25 expression and elevated ROS compared to CD4^+^ T cells cultured alone (Figure 7H,I and Figure S15). The cocultured CD4^+^ T cells also exhibited increased death (Figure 7J) consistent with multimodal PANoptosis encompassing the activation of Caspase‐1/3/8, GSDMD, MLKL and RIPK3 (Figure 7K). Moreover, intracellular succinate accumulation only occurred in CD4^+^ T cells co‐cultured with monocytes, but not those cultured alone, in high‐glucose conditions (Figure S16), suggesting that CD4^+^ T cells acquire exogenous succinate released by monocytes. To further address the role of SDH in the hyperglycemia‐induced succinate accumulation, monocytes were primed with the SDH inhibitor Atpenin A5 before co‐culture with CD4^+^ T cells in normal or high‐glucose conditions. In the normal glucose condition, Atpenin A5 priming greatly increased succinate level and death of CD4^+^ T cells, suggesting the critical role of SDH in the accumulation of succinate and cell death (Figure S17A,B). However, high‐glucose treatment did not further increase intracellular succinate accumulation and death of CD4^+^ T cells after Atpenin A5 priming (Figure S17C,D). These findings strongly indicate that monocyte SDH impairment by elevated glucose concentrations drives succinate accumulation and release and causes CD4^+^ T cell death.

## 3 Discussion

**FIGURE 8:**
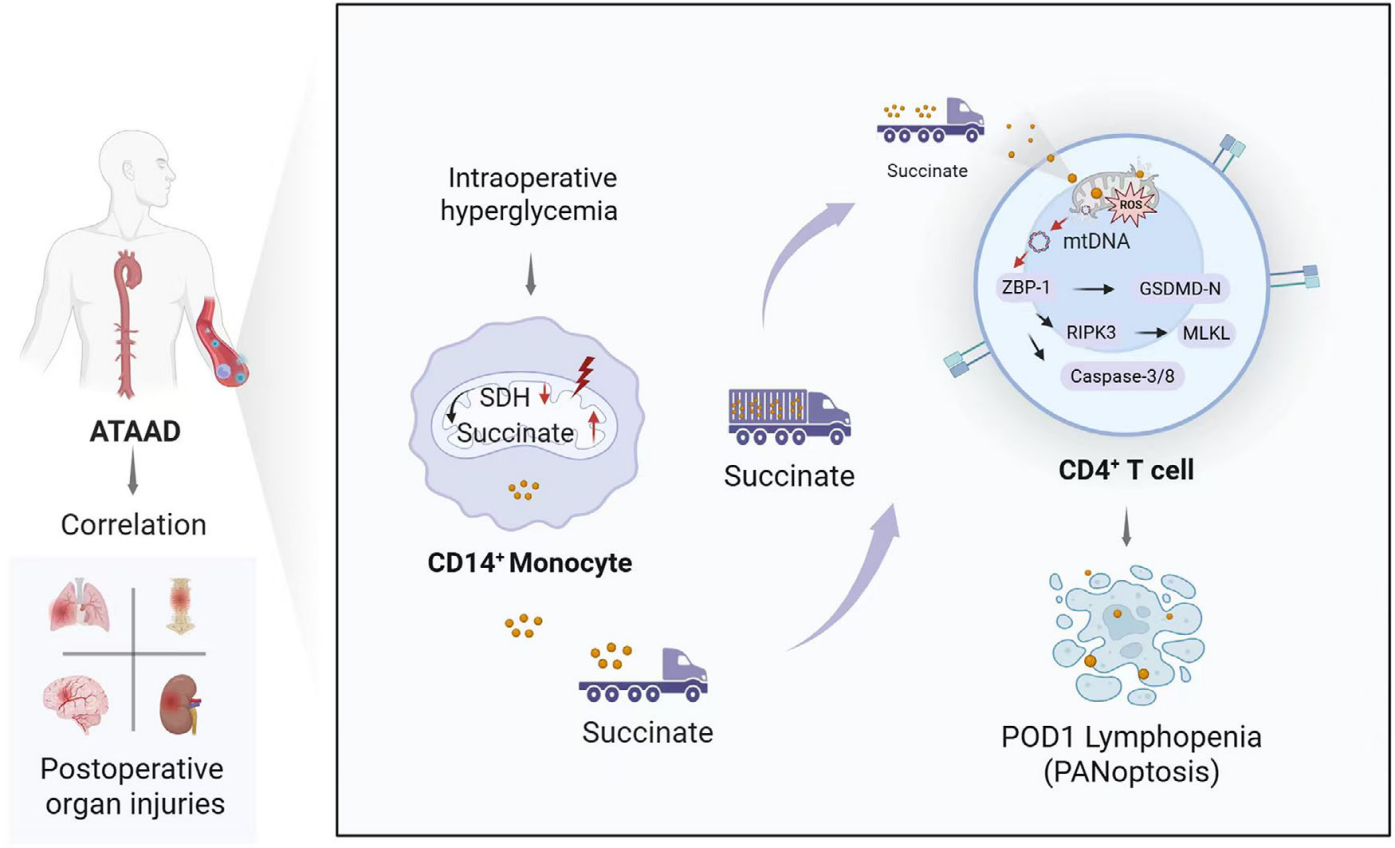
Mechanistic model of monocyte‐CD4^+^ T cell crosstalk mediating high glucose‐induced PANoptosis and POI in patients undergoing major surgery. Intraoperative hyperglycemia promotes PANoptosis in CD4^+^ T cells and is linked to postoperative organ injury (POIs) in patients undergoing total aortic arch replacement. High glucose suppresses succinate dehydrogenase (SDH) in monocytes, leading to succinate accumulation and release; succinate then drives mitochondrial reactive oxygen species (ROS) production and mtDNA release in CD4^+^ T cells. The liberated mtDNA activates intracellular Z‐DNA binding protein 1 (ZBP1), triggering inflammatory PANoptosis and disrupting CD4^+^ T cell homeostasis, thereby contributing to POIs.

How stress hyperglycemia contributes to postoperative organ injury remains poorly understood. Our work in this study finds that stress hyperglycemia independently predicts adverse postoperative outcomes and is strongly associated with lymphopenia early after surgery, with the most substantial reduction of CD4^+^ T cells. The rapid and profound loss of CD4^+^ T cells results predominantly from inflammatory PANoptosis, a form of multimodal cell death previously described largely in innate immune cells. CD4^+^ T cell PANoptosis is triggered by a cellular crosstalk involving elevated glucose driving succinate accumulation and release from monocytes to compromise the mitochondrias of CD4^+^ T cell which activates the ZBP1 pathway of PANoptosis. Such a previously unappreciated monocyte–T cell cascade links hyperglycemic distress to immune dysfunction that contributes to postoperative organ injury (Figure 8).

Mounting evidence indicates that the prognostic impact of perioperative hyperglycemia differs fundamentally between patients with and without diabetes [1, 29]. Randomized trials have shown that intensive glucose control confers no clear benefit in those with established diabetes, likely due to chronic metabolic adaptation and altered glucose–insulin responsiveness [30]. In contrast, nondiabetic patients have substantially reduced postoperative complications and mortality under intensive glycemic management, suggesting that stress‐induced hyperglycemia reflects acute metabolic and immune dysregulation rather than a manifestation of chronic glycemic tolerance [31, 32]. This divergence underscores stress hyperglycemia as not merely a transient metabolic phenomenon but a potent trigger of immune dysfunction that drives POIs. Elucidating the adaptive and maladaptive immune mechanisms driven by stress hyperglycemia is therefore essential for identifying vulnerable individuals and developing targeted perioperative strategies.

The detrimental influence of perioperative hyperglycemia on immune function and subsequent risk has been previously documented. For instance, postoperative hyperglycemia was reported to markedly attenuate cytokine production in whole‐blood cultures following mitogenic stimulation, suggesting a suppression of cell‐dependent immune activation under hyperglycemic conditions [33]. Building upon this correlation, insights from our study can lead to a more comprehensive and mechanistic resolution to the question of how sustained stress hyperglycemia ultimately drives immune dysfunction. Through integrated metabolomic and immunological analyses, this novel immunometabolic pathway of hyperglycemia‐driven monocyte metabolic perturbation involving succinate buildup and release that causes lymphocyte death via the release of mtDNA not only can explain the lymphocyte loss long observed clinically but also provides a molecular basis for the adverse outcomes seen in patients with major surgical trauma, thus establishing stress hyperglycemia as a critical and actionable determinant of postoperative immune imbalance and organ injury.

The immune implications of this monocyte–succinate–CD4^+^ T cell PANoptosis pathway are broad. By identifying PANoptosis as a mode of CD4^+^ T cell death under hyperglycemic stress, our study extends the relevance of PANoptosis, previously described mainly in innate immune cells during infection and malignancy, to metabolic lymphopenia after surgical trauma. Metabolic lymphopenia is a common feature of many acute and chronic inflammatory conditions, including sepsis, major surgical trauma, HIV infection, and diabetes. Although the regulation and functional consequences of PANoptosis in these settings remain incompletely defined, microbial and damage‐associated molecular patterns may promote PANoptosome assembly and initiate PANoptotic cell death, suggesting that PANoptosis may represent a shared mechanism of immune‐cell loss under metabolic and inflammatory stress.

Notably, the postoperative CD4^+^ T‐cell loss observed in our study appears mechanistically distinct from classical T‐cell exhaustion. While both chronic exhaustion and acute metabolic stress can ultimately lead to T‐cell loss or dysfunction [34, 35], the CD4^+^ T‐cell phenotype observed in our study developed rapidly after surgery and was characterized by mitochondrial injury, inflammatory cytokine release, and direct activation of PANoptotic cell‐death pathways. Thus, although prolonged hyperglycemia or persistent postoperative inflammation may engage exhaustion‐related circuits over a longer timeline, our current findings suggest that this severe early decline in CD4^+^ T cells is more likely driven by an acute PANoptotic program rather than a classical chronic exhaustion process.

Our data that the combined metabolic stress of surgical trauma and hyperglycemia elevates monocyte‐derived succinate aligns with current evidence that monocytes and macrophages accumulate or secrete succinate to promote their own proinflammatory activation as well as that of other neighboring or systemic immune cells [36, 37]. Together with the study showing succinate uptake impairs T cell mitochondrial metabolism by reducing TCA cycle flux [38], our finding underscores the concept that T cells are sensitive detectors and interpreters of metabolic distress of other immune cells. Emerging evidence also suggests that inflammatory cytokines and metabolic stress can induce antigen‐independent “bystander activation” of T cells and sensitize them to activation‐associated programmed cell death pathways through stress‐responsive signaling mechanisms [39, 40, 41, 42]. Consistent with this concept, the increased activation state observed in hyperglycemia‐exposed CD4^+^ T cells in our study are more likely attributable to stress‐associated inflammatory signaling rather than rather than conventional antigen‐driven activation. Such a bystander‐like activation state may render CD4^+^ T cells more vulnerable to PANoptotic signaling in the presence of metabolic stress.

Recent studies have demonstrated that hyperglycemia can induce trained immunity in monocytes through sustained metabolic and epigenetic reprogramming, resulting in a persistent pro‐inflammatory phenotype even after glucose levels normalize [43, 44]. In this context, the metabolic remodeling of monocytes observed in our study may not only contribute to CD4^+^ T‐cell dysfunction but also predispose infiltrating monocytes/macrophages to a pro‐inflammatory program, thereby exacerbating tissue injury in vulnerable organs such as the lung, kidney, and spinal cord [45, 46, 47]. Such a mechanism may represent an additional link between stress hyperglycemia and postoperative organ injury. The concept of trained immunity may also help identify vulnerable patients at increased risk of postoperative immune dysregulation and organ injury.

Several limitations of this study should be acknowledged. First, although our findings support a causal link between stress hyperglycemia, monocyte‐derived succinate accumulation, and CD4^+^ T cell PANoptosis, in vivo studies of post‐surgery patients and mouse models are needed to confirm and elucidate the contribution of this pathway to various forms of POI. Second, the study focused mainly on circulating immune cells, whereas tissue‐resident immune cells, such as hepatic and pulmonary tissue‐resident macrophages or T cells, may also undergo PANoptosis in response to hyperglycemia and contribute to local inflammatory injury. Third, the precise succinate transport or sensing pathways in CD4^+^ T cells were not further dissected in the present study, mainly because these mechanistic assays in primary human CD4^+^ T cells require stable ex vivo metabolic conditions, reliable experimental manipulation, and substantial numbers of highly viable clinical cells. Fourth, while we controlled for key perioperative variables, our retrospective design remains subject to residual confounding from unobserved factors, such as intraoperative catecholamine use. Consequently, further validation in prospective studies is warranted. Finally, although our data reveal strong associations between hyperglycemia and immune dysfunction including lymphopenia in acute surgical stress, prospective interventional trials are required to determine whether targeted glycemic or metabolic modulation can effectively prevent immune dysregulation and improve postoperative outcomes.

In conclusion, our findings define a previously unrecognized immunometabolic cascade linking surgical trauma and stress hyperglycemia to POI. This cascade contributes to postoperative lymphopenia and immune dysregulation and establishes T cell PANoptosis as a clinically significant consequence of stress and possibly other causes that converges on metabolic alterations and immune paralysis. Further understanding of this monocyte–T cell metabolic crosstalk will provide mechanistic insights for identifying high‐risk patients susceptible to POI or conditions with a shared immunopathogenic mechanism who can benefit from the targeting of hyperglycemia‐induced immunometabolic stress.

## 4 Materials and Methods

### 4.1 Patient Population

We collected data on patients with ATAAD undergoing open surgical repair between April 2017 and April 2025 at two tertiary centres in China: The Second Xiangya Hospital and Xiangya Hospital, Central South University. The study was approved by the institutional review board of The Second Xiangya Hospital (LYEC2024‐K0033), registered in the Chinese Clinical Trial Registry (ChiCTR2500103899), and conducted in accordance with the Declaration of Helsinki. The requirement for written informed consent was waived by the institutional review board.

### 4.2 Inclusion and Exclusion Criteria

The study included all ATAAD patients who underwent open repair surgery. Of the 492 identified ATAAD patients, those with diabetes or missing intraoperative blood glucose (BG) data were excluded, resulting in 370 patients in the final cohort (Figure S1). The inclusion criteria constituted all patients undergoing open repair surgery for Stanford type‐A aortic dissection. Exclusion criteria encompassed pregnancy, insufficient pre‐operative data, non‐acute dissection, concurrent severe condition (e.g., infection, cancers, and immune deficiency), preoperative organ injury, and diabetes, which was excluded to allow a more specific evaluation of acute stress hyperglycemia independent of chronic dysglycemia‐related metabolic and immune alterations.

### 4.3 Anesthesia and Surgical Procedures

A standardized perioperative management protocol was implemented across both participating centers, consistent with previously described procedures [48, 49]. Briefly, general anesthesia was induced with propofol, sufentanil, vecuronium, and sevoflurane, and maintained via continuous infusion of propofol and atracurium for skeletal muscle relaxation. Arterial cannulation was preferentially performed via the left axillary artery, with the femoral artery serving as an alternative access site in cases of dissection involvement or elevated pump pressures. Upon achieving the target core temperature, antegrade cerebral perfusion was initiated. Systemic perfusion was restored following completion of the proximal anastomosis, concomitant with the initiation of rewarming. During the rewarming phase, reconstruction of the supra‐aortic branch vessels was performed. Postoperatively, all patients were transferred to the intensive care unit (ICU) while remaining intubated for further management.

### 4.4 Data and Patient Specimen Collection

Data were obtained from the electronic medical records of each hospital. Collected variables included patient demographics, medical history, and postoperative organ injury. Intraoperative data such as mechanical ventilation time, ICU stay length, and hospital stay length were also recorded.

Hyperglycemia is defined as any BG value ≥140 mg/dL [26, 50]. Intraoperative BG measurements were taken hourly. After surgery, BG concentrations were assessed every 4 h for the first 24 h during the ICU stay. The mean BG was calculated as the average of all recorded glucose values, while the peak BG represented the highest value observed during the monitoring period [51]. Peripheral blood samples were collected from ATAAD patients preoperatively and 24 h after surgery.

### 4.5 Assessment of Postoperative Organ Injury

Postoperative organ injury refers to damage to key organs, including the brain, lungs, liver, spinal cord, and kidneys, that occurs following surgery in patients who have no pre‐existing organ dysfunction. The definition criteria for organ injury are referenced from published papers [12, 52, 53, 54, 55, 56, 57, 58, 59]. Brain injury is defined as any cerebrovascular event, such as transient ischemic attacks, stroke, or cerebral hemorrhage, diagnosed through clinical assessment and neuroimaging [54]. Lung injury is defined as the postoperative occurrence of the following conditions, including mild respiratory failure, severe respiratory failure, pleural effusion, atelectasis, respiratory infection, pneumothorax, bronchospasm, and aspiration pneumonitis [55]. Liver injury is defined as any postoperative serum ALT value measured after surgery exceeded five times the upper limit of normal (ULN) [57]. Postoperative spinal cord injury was defined as any new lower extremity motor and/or sensory deficit after surgery. Lower extremity motor function was classified according to the modified Tarlov scale [58]. Postoperative AKI was diagnosed based on the Kidney Disease Improving Global Outcomes (KDIGO) criteria, which include an absolute increase in serum creatinine of ≥0.3 mg/dL or a ≥50% increase in serum creatinine within 7 days after surgery [59].

### 4.6 Cell Isolation and in Vitro Culture

Different cell populations were isolated from fresh EDTA‐anticoagulated human blood for functional assays. Peripheral blood mononuclear cells (PBMCs) were first isolated by Ficoll‐Hypaque density gradient centrifugation. Monocytes and CD4^+^ T cells were then sequentially purified from the PBMC fraction using the EasySep Direct Human Monocyte Isolation Kit (STEMCELL Technologies, 19669) and the EasySep Direct Human CD4^+^ T Cell Isolation Kit (STEMCELL Technologies, 19662), respectively, according to the manufacturers' protocols. Flow cytometric analysis confirmed that the purity of the resulting CD14^+^ monocyte and CD4^+^ T cell populations routinely exceeded 95% (Figure S12).

For depletion studies, CD4^+^ T cells or CD14^+^ monocytes were removed from PBMCs using the Human CD4 Microbeads kit (Miltenyi Biotec, 130‐045‐101) or the Human CD14 Microbeads kit (Miltenyi Biotec, 130‐097‐052), respectively, with separation over an LD column. Post‐depletion analysis revealed that 0.2% or less of the targeted cells remained [60]. All cells were cultured in complete RPMI‐1640 medium, supplemented with 10% fetal bovine serum and 1% penicillin‐streptomycin, and maintained at 37°C in a 5% CO_2_ humidified atmosphere until use.

For the experiments in Figures 2, 3, 4, 5, PBMCs, isolated CD4^+^ T cells, or CD4^+^ T cell‐depleted PBMCs were cultured for 24 h in RPMI‐1640 medium containing 1% penicillin‐streptomycin, the patient's autologous serum, and specific stimuli (e.g., high glucose or succinate). For Figure 6, isolated CD4^+^ T cells, CD14^+^ monocytes, CD4^+^ T cell‐depleted PBMCs, or CD14^+^ monocyte‐depleted PBMCs were treated for 24 h with high glucose, succinate, or mitochondrial DNA. For Figure 7, direct co‐cultures of CD4^+^ T cells and CD14^+^ monocytes were established under high‐glucose conditions. All cultures were maintained at 37°C in a 5% CO_2_ atmosphere. A complete list of reagents is provided in Table S5.

### 4.7 Flow Cytometry

For surface marker staining, PBMCs were incubated with fluorochrome‐conjugated monoclonal antibodies for 30 min at 4°C in the dark. The following antibodies were used: anti‐CD45 (Biolegend, 368515, APC‐Cy7), anti‐CD3 (Biolegend, 300419, PE/Cy7), anti‐CD4 (Biolegend, 357413, PerCP/Cy5.5), anti‐CD8 (BD Pharmingen, 563256, BV510), and anti‐CD14 (BD Pharmingen, 563743, BV421). Cells were then washed twice with FACS buffer (PBS supplemented with 2% fetal bovine serum) and resuspended in the same buffer for acquisition. To determine absolute cell counts, a known quantity of fluorescent counting beads (Biolegend,424902) was added to each sample immediately before acquisition on a flow cytometer.

For T cell activation assays, PBMCs were stimulated with 30 µg/mL phytohemagglutinin (PHA; InvivoGen, R30852801) for 24 h at 37°C under 5% CO_2_. Cells were then stained for CD3 (Biolegend, 300419, PE/Cy7), CD4(Biolegend, 357413, PerCP/Cy5.5), CD69 (BioLegend, 310903, FITC), and CD25 (BD Pharmingen, 567316, APC) to quantify activation as the percentage of CD69^+^ and/or CD25^+^ cells within the CD3^+^CD4^+^ T lymphocyte population.

Cell viability and apoptosis were assessed using an Annexin V‐fluorochrome/7‐AAD kit (Biolegend, 640922) according to the manufacturer's instructions. Viable (Annexin V^−^/7‐AAD^−^), and late apoptotic/dead (Annexin V^+^/7‐AAD^+^) cell populations were distinguished. Detailed information on antibodies, clones, and staining panels is provided in Table S6 and S7.

### 4.8 Mitochondrial Function

Mitochondrial function was assessed in CD4^+^ T cells following specific stimulation. The cells were either first isolated from PBMCs and then stimulated, or stimulated within the PBMCs population and subsequently stained for CD3 (Biolegend, 300419, PE/Cy7) and CD4 (Biolegend, 357413, PerCP/Cy5.5). General and mitochondrial ROS levels were measured by flow cytometry using DCFH‐DA and MitoSOX Red (ThermoFisher, 88‐5930‐74), respectively, and mitochondrial membrane potential (ΔΨm) was quantified with the JC‐10 assay kit (Biolegend, 421902). Additionally, intracellular ATP levels were determined from 1 × 10^6^ purified and stimulated CD4^+^ T cells using a commercial ATP assay kit (Abcam, ab83355). All commercial kits were used according to the manufacturers' protocols.

### 4.9 RNA‐Sequencing (RNA‐seq) and Bioinformatics Analysis

RNA sequencing (E‐MTAB‐16247) was performed on CD14^+^ monocytes isolated from ATAAD patients and cultured under either normal glucose (5.5 mM, n = 4) or high glucose (30 mM, n = 4) conditions for 24 h. RNA‐seq library construction and sequencing were carried out on the Illumina platform by Genedenovo Biotechnology Co., Ltd. (Guangzhou, China), following standard protocols as described in previous studies [11, 55]. Differentially expressed genes (DEGs) were identified using DESeq with a fold change >1.5 and a q‐value < 0.05. Gene set enrichment analysis (GSEA) was performed with clusterProfiler based on MSigDB hallmark and KEGG gene sets, ranking genes by log_2_ fold change [12, 13].

### 4.10 Serum Metabolomics Analysis

Serum samples were collected at the start of surgery and on postoperative day 1, then immediately stored at −80°C until analysis. For metabolite extraction, 100 µL of serum was mixed with 400 µL of ice‐cold methanol: acetonitrile (1:1), vortexed for 30 s, and incubated at −20°C for 30 min to precipitate proteins. The mixture was then centrifuged at 12,000 g for 10 min at 4°C, and the supernatant was filtered through a 0.22 µm filter. Liquid chromatography‐mass spectrometry (LC‐MS) was performed in both positive and negative ion modes. Data were processed using appropriate software for peak identification and quantification. Quality control samples were included to ensure the reliability and reproducibility of the analysis.

### 4.11 Real‐Time Quantitative PCR (RT‐qPCR)

Total RNA was extracted from CD4^+^ T cells with TRIzol Reagent (Thermo Fisher, 15596026) following the manufacturer's instructions. Subsequently, cDNA was synthesized using the RevertAid First Strand cDNA Synthesis Kit (ThermoFisher, K1622). Real‐time PCR was carried out with Fast SYBR Green Master Mix (Novoprotein, E301) on a CFX96 Touch Deep‐Well Real‐Time PCR Detection System (Bio‐Rad Laboratories, Inc.). Gene expression levels were quantified via the 2^−ΔΔCT^ method, and the corresponding primer sequences are provided in Table S8.

### 4.12 ELISA

Cytokine levels of IL‐1β, IL‐6, and TNF were measured using the Human IL‐1β Quantikine ELISA Kit (R&D Systems, DY201), Human IL‐6 Quantikine ELISA Kit (R&D Systems, D6050), and Human TNF Quantikine ELISA Kit (R&D Systems, DTA00D) according to the manufacturers' instructions. The assays were performed on lysates from 1 × 10^6^ CD4^+^ T cells for fold‐change comparison and on serum samples for absolute quantification.

### 4.13 Western Blot

Magnetic bead sorting was applied to isolate the target cell populations (CD4^+^ T cells or CD14^+^ monocytes) from respective conditions, followed by western blot (WB) analysis on the sorted cells. The sorted cells were lysed in RIPA buffer supplemented with phosphatase protease inhibitors and protease inhibitors. Proteins were separated by SDS‐PAGE and transferred onto polyvinylidene difluoride (PVDF) membranes. After blocking with 5% non‐fat milk for 30 min at room temperature, the membranes were incubated overnight at 4°C with the following primary antibodies: caspase‐3 (Cell Signaling Technology, #9662, 1:1000), cleaved caspase‐3 (Cell Signaling Technology, #9664, 1:1000), caspase‐8 (Cell Signaling Technology, #4790, 1:1000), cleaved caspase‐8 (Proteintech, 85171‐3‐RR, 1:2000), caspase‐1 (Proteintech, 84735‐1‐RR, 1:2000), cleaved caspase‐1 (Cell Signaling Technology, #4199, 1:1000), GSDMD‐N (Abcam, ab215203, 1:1000), GSDMD (Abcam, ab219800, 1:2000), MLKL (Cell Signaling Technology, #14993, 1:1000), pMLKL (Cell Signaling Technology, #91689, 1:1000), RIPK3 (Cell Signaling Technology, #13526, 1:1000), pRIPK3 (Cell Signaling Technology, #93654, 1:1000), ZBP1 (Proteintech, 13285‐1‐AP, 1:1000), SDHB (Proteintech, 10620‐1‐AP, 1:500), β‐actin (Proteintech, 81115‐1‐RR, 1:5000). Following incubation with horseradish peroxidase (HRP)‐conjugated secondary antibodies (Cell Signaling Technology,7074), protein bands were visualized using a chemiluminescent substrate and quantified by densitometry.

### 4.14 Co‐Immunoprecipitation Assays (Co‐IP)

For co‐immunoprecipitation assays, CD4^+^ T cells isolated from human PBMCs were subjected to treatment with 5.5 mM or 30 mM glucose for 24 h, or with PBS or 10 mM succinate for 24 h; additionally, POD1 CD4^+^ T cells from patients stratified by the duration of intraoperative blood glucose ≥140 mg/dL or postoperative lymphopenia on POD1. After treatment, cells were washed with PBS, harvested, and lysed in IP Lysis Buffer (Thermo Fisher, 87788) supplemented with phosphatase and protease inhibitors on ice for 30 min. Lysates were centrifuged at 14,000 × g for 15 min at 4°C, and supernatants were collected, with 5% of each supernatant reserved as input control and stored at −80°C. The remaining supernatants were incubated overnight at 4°C with protein A/G magnetic beads (Selleck, B23201) along with either an anti‐RIPK3 antibody (Santa Cruz Biotechnology, sc‐374639) or a control IgG. Immunoprecipitated complexes were washed and boiled in 1× SDS loading buffer at 100°C for 10 min, and precipitated proteins were separated by SDS‐PAGE, transferred to PVDF membranes, and analyzed by immunoblotting using anti‐NLRP3 (Cell Signaling Technology, #15101, 1:1000), anti‐RIPK3 (Cell Signaling Technology, #13526, 1:1000), anti‐caspase‐8 (Cell Signaling Technology, #4790, 1:1000), anti‐ZBP1 (Cell Signaling Technology, #60968, 1:1000), anti‐ASC (Proteintech, 10500‐1‐AP, 1:1000), and anti‐β‐actin (Proteintech, 81115‐1‐RR, 1:5000) antibodies.

### 4.15 Succinate Colorimetric Assay

The succinate colorimetric assay is used to measure succinate levels within 24 h post‐surgery. Succinate concentration is determined through a coupled enzyme reaction, which produces a colorimetric product detectable at 450 nm. The absorbance is proportional to the succinate concentration in the sample. Detailed procedures follow the instructions provided by succinate assay kit (Sigma‐Aldrich, MAK335).

### 4.16 Transmission Electron Microscopy

Transmission electron microscopy was performed to assess mitochondrial ultrastructure. CD4^+^ T cells were fixed with 2.5% glutaraldehyde in 0.1 M Sorenson's buffer for 3 h, followed by post‐fixation with 1% OsO_4_ in the same buffer for 2 h. After en bloc staining with 1% tannic acid and dehydration through a graded ethanol series, samples were embedded in a resin mixture of Lx‐112 and Embed‐812. Ultrathin sections were stained with 1% uranyl acetate and 0.4% lead citrate and examined using a TEM‐1400 Plus electron microscope. For quantitative analysis, ten CD4^+^ T cells per sample were randomly selected and imaged at ×50,000 magnification [12, 13].

Mitochondrial numbers were counted manually, and mitochondrial length was measured by ImageJ software. Damaged mitochondria were identified by loss of cristae and/or broken mitochondrial outer and/or inner membrane [61].

### 4.17 mtDNA Preparation

Mitochondrial DNA (mtDNA) was isolated from THP‐1 cells by first enriching mitochondria and then purifying mtDNA following the approach described by Christian Frezza and colleagues [62, 63]. Briefly, mitochondria were purified from cytosol/nuclei using a mitochondrial isolation kit (Thermo Fisher, 89874) according to the manufacturer's instruction. Subsequently, the freshly isolated mitochondria were subjected to mtDNA extraction using the mtDNA isolation kit (Abcam, ab65321) under nuclease‐free conditions. The purity of the isolated mtDNA was verified by qPCR for mitochondrial (e.g., MT‐ND1) versus nuclear (e.g., B2M/β‐actin) markers.

### 4.18 mtDNA Transfection

Transfection of mtDNA into CD4^+^ T cells was performed using Lipofectamine 2000 (Thermo Fisher, 11668019). Briefly, activated CD4^+^ T cells were harvested and resuspended in antibiotic‐free medium. For each transfection, 1 µg of purified mtDNA was mixed with the transfection reagent in serum‐free medium, incubated for 20 min to form complexes, and then added to the cells. After 6–8 h of incubation at 37°C, the transfection mixture was replaced with fresh complete medium. The cells were further cultured for 24–48 h prior to experimental treatment.

### 4.19 mtDNA Copy Quantification

Supernatants and serum samples were centrifuged (300 × g, 5 min; then 2,000 × g, 10 min at 4°C) to remove debris, followed by 0.22 µm filtration. Cell‐free DNA was extracted using the mtDNA isolation kit (Abcam, ab65321). The absolute mitochondrial DNA copy number was quantified by quantitative PCR (RT–qPCR) using the Absolute Human Mitochondrial DNA Copy Number Quantification qPCR Assay Kit (Sciencell, 8948) [64].

### 4.20 Statistical Analysis

Statistical analyses were performed using GraphPad Prism (v8). Continuous variables are expressed as mean ± SEM or median (IQR), as appropriate, while categorical variables are presented as numbers and percentages. For comparisons between two groups, the Student's *t*‐test or Mann‐Whitney U test was employed, depending on data distribution. Comparisons among multiple groups were conducted using one‐way ANOVA with Tukey's post‐hoc test for normally distributed data. Categorical data were analyzed using the χ^2^ test or Fisher's exact test as applicable. Multivariable logistic regression analyses were performed to identify independent factors associated with postoperative organ injuries and postoperative day 1 lymphopenia. Variables with *p* < 0.20 in univariable analyses were entered into the multivariable models, and adjusted odds ratios (ORs) with 95% confidence intervals (CIs) were reported. Correlations between CD4^+^ T cell subsets and clinical outcomes were assessed using Pearson correlation. *p*‐value < 0.05 was considered statistically significant.

## Author Contributions

S.Z. and X.M.M. contributed equally to this work. Conceptualization: S.Z., and X.M.M. Methodology: X.M.M., W.L., H.L., T.L., W.Y.S.Z., and Y.L. Investigation: S.Z., and X.M.M. Data curation: X.M.M., J.Y. L, and D.F. Software: T.L., and Y.W. Validation: S.Z., Y.W., and W.Y.S. Formal analysis: S.Z., X.M.M., J.Y. L. Visualization: S.Z., X.M.M. Supervision: R.P.D., H.L., and W.L. Funding acquisition: T.L., X.M.M., H.L., S.Z. and R.P.D. Project administration: H.L., and R.P.D. Resources: H.L., and R.P.D. Writing – original draft: H.L., and R.P.D. Writing – review & editing: H.L., R.P.D., K.C, S.Z. and X.M.M. All authors made significant contributions to the work and approved the final version for submission.

## Conflicts of Interest

The authors declare no conflicts of interest.

## Supporting information




**Supporting File 1**: advs76968‐sup‐0001‐SuppMat.pdf.

**Supporting File 2**: advs76968‐sup‐0002‐Data.zip.

## Data Availability

The data that support the findings of this study are openly available in ArrayExpress at https://www.ebi.ac.uk/biostudies/ArrayExpress/studies/E‐MTAB‐16247, reference number E‐MTAB‐16247.
