# Supplementary material for: Stress Hyperglycemia Drives CD4+ T Cell PANoptosis and Postoperative Organ Injury via Monocyte‐Derived Succinate
Source: Adv Sci (Weinh). 2026 Aug 5;13(60):e76968. doi: 10.1002/advs.76968 (PMC13440196; doi:10.1002/advs.76968)
Supplement: Supplementary file 1 [file ADVS-13-e76968-s002.pdf]

Stress hyperglycemia drives CD4$^+$ T cell PANoptosis and postoperative organ injury via monocyte-derived succinate

Shuai Zhao, Xuemei Miao *et al.*

*Corresponding author. Email: Ru-Ping Dai, xyeyyrupingdai@csu.edu.cn; Hui Li, lihui_1166@csu.edu.cn

This PDF file includes:

Figures S1 to S17
Tables S1 to S8

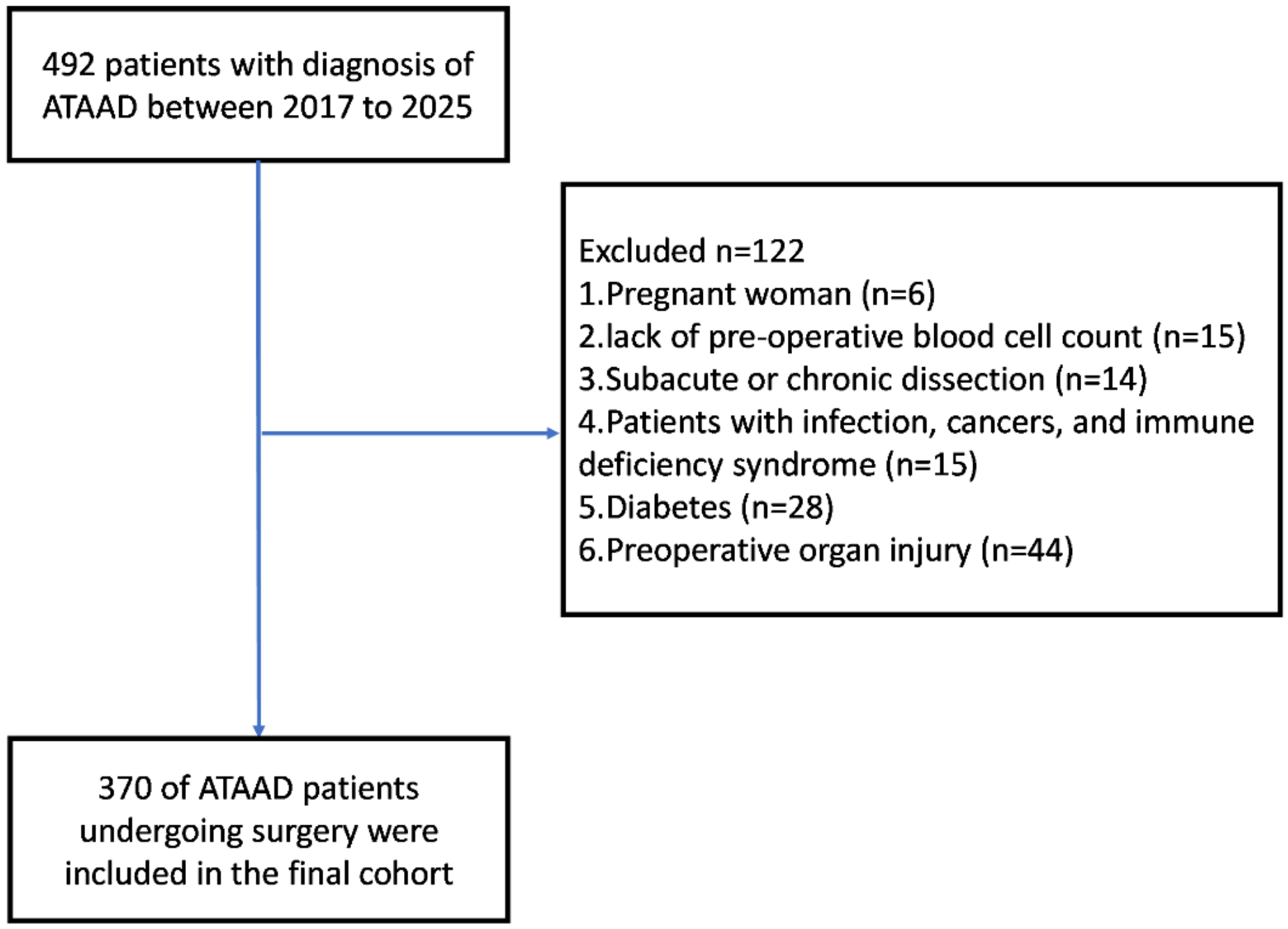

Figure S1. Inclusion criteria for ATAAD study cohort.
Out of 492 ATAAD patients assessed from 2017 to 2025, 122 were excluded due to pregnancy, insufficient pre-operative data, non-acute dissection, concurrent severe conditions, diabetes, and postoperative glucose data loss. The remaining 370 patients were included in the final surgical cohort.

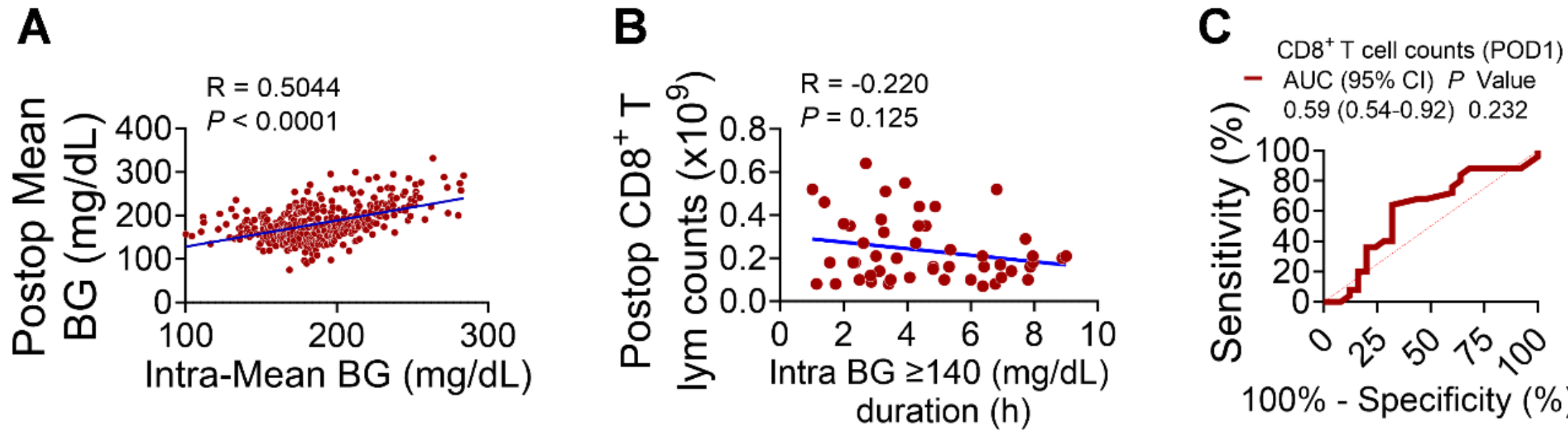

Figure S2. Correlation and predictive analyses of blood glucose and CD8⁺ T cells for postoperative outcomes.

A) Correlation analysis of intraoperative mean blood glucose and postoperative (Postop) mean blood glucose from ATAAD (n = 370). **B**) Correlations between postoperative $CD8^+$ T cells counts and the duration of intraoperative (Intra) blood glucose (BG)⩾140 mg/dL (n=50). **C**) ROC analysis assessing the predictive performance of postoperative day 1 (POD1) $CD8^+$ T-cells counts for POIs. Data are presented as median (IQR) or mean ± SEM. Statistical analyses were performed using Pearson correlation (**A, B**), and ROC analysis with AUC values (DeLong 95% CI; **C**).

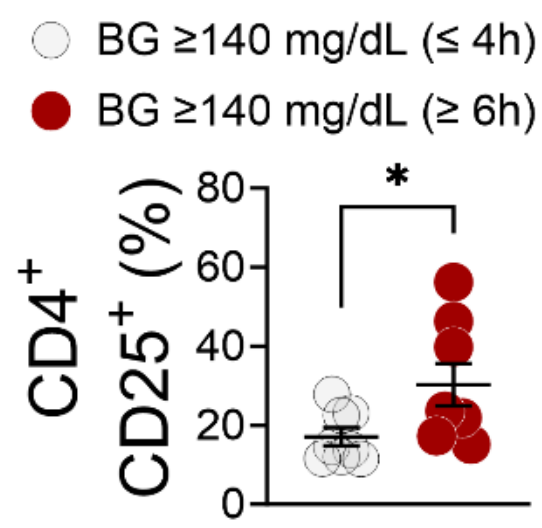

Figure S3. Assessment of CD25^{+} CD4^{+} T cells stratified by intraoperative hyperglycemia duration.

CD25^{+} cells among circulating CD4^{+} T cells were analyzed by flow cytometry in patients stratified according to intraoperative hyperglycemia duration, defined as BG ≥140 mg/dL for ≤4 h or ≥6 h. Each point represents an individual patient. Data are presented as mean ± SEM. Statistical significance was determined using Student's t-test. *$P < 0.05$.

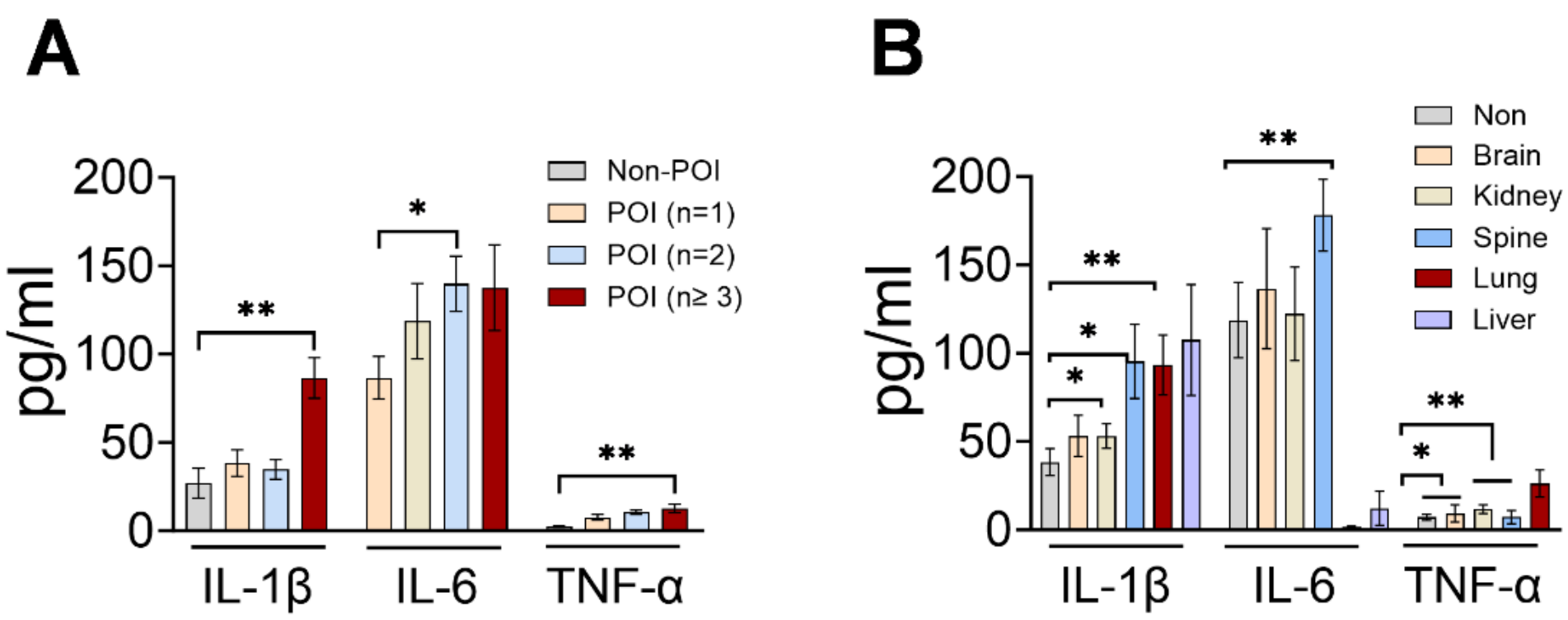

Figure S4. Serum inflammatory cytokine levels are stratified by the number and type of POIs in ATAAD patients.

Serum samples were collected on postoperative day 1 (POD1) to measure inflammatory cytokines and proteins from ATAAD. **A**) ELISA measurements of serum inflammatory cytokines (IL-1β, IL-6 and TNF-α) 24 hours after surgery in patients with different numbers of postoperative organ injuries (POIs) (n = 6 per group). **B**) Comparison of inflammatory cytokine levels across patients with specific organ injuries (n = 3-7 per group). Data are presented as mean ± SEM. One-way ANOVA was used for statistical analysis. *$P < 0.05$; **$P < 0.01$.

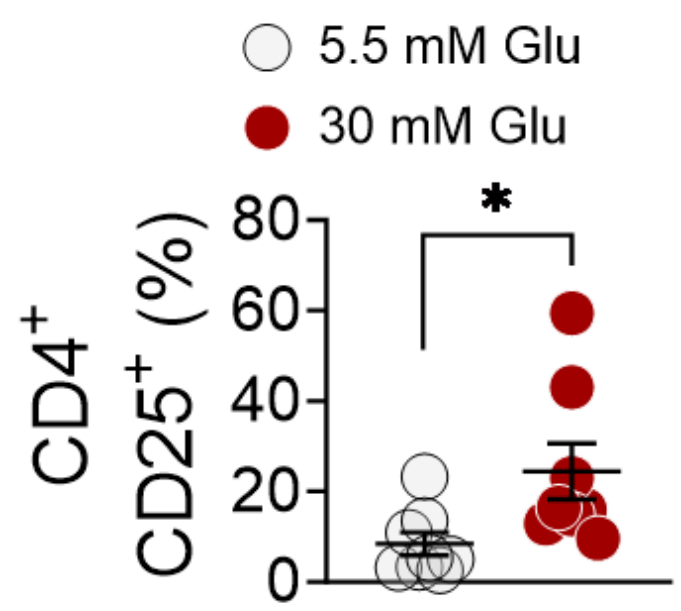

Figure S5. Effects of high glucose on CD4+ T cell activation.
PBMCs from ATAAD patients were cultured for 24 h in media containing either 5.5 mM or 30 mM glucose. Flow cytometric analysis of $CD25^{+}$ $CD4^{+}$ T cell proportions was performed to assess T cell activation. Each point represents an individual patient. Data are presented as mean ± SEM. Statistical significance was determined using Student's t-test. *$P < 0.05$.

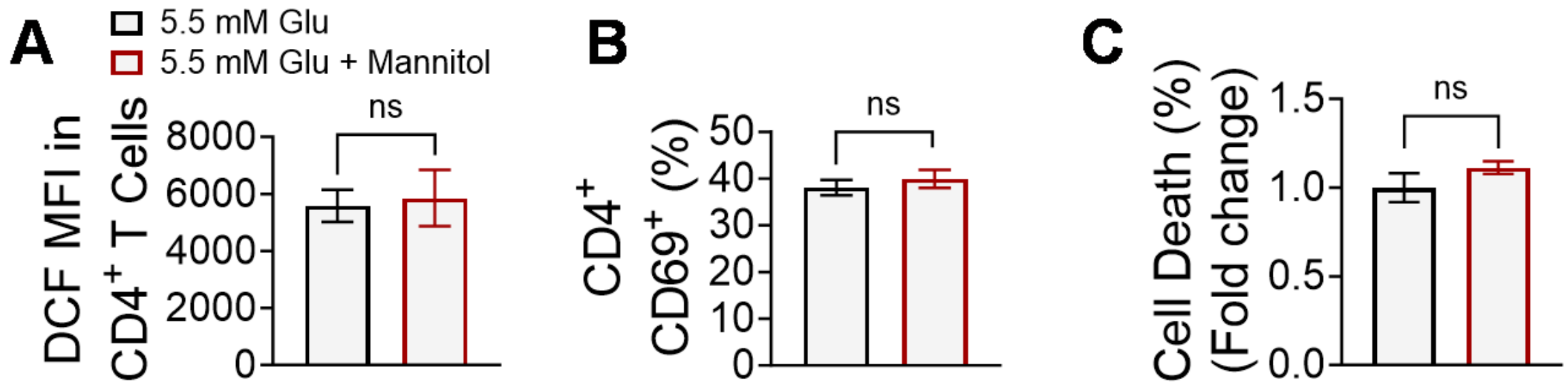

Figure S6. Effects of isotonic conditions on $CD4^+$ T cell function and viability. PBMCs from ATAAD patients were cultured for 24 h in media containing 5.5 mM glucose or an osmotic control (5.5 mM glucose + 24.5 mM mannitol). **A-C**) Flow cytometry was used to assess $CD4^+$ T cells for DCF MFI (**A**), cell activation (**B**), and cell death (**C**) (n = 6 per group). Data are presented as mean ± SEM. Student's t-test were used for statistical analysis. ns not significant.

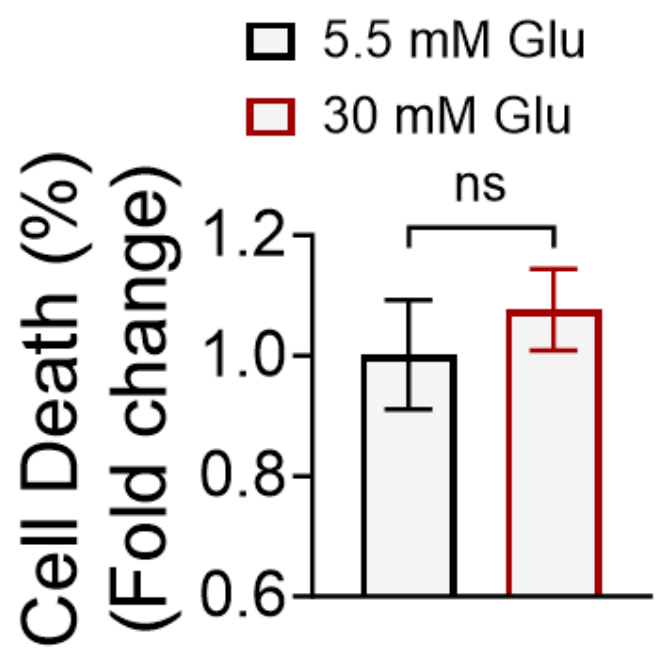

Figure S7. Effects of high glucose on $CD4^+$ T cell viability.
$CD4^+$ T cells were magnetically isolated from ATAAD patients cultured for 24 h in media containing either 5.5 mM or 30 mM glucose (Glu). Flow cytometry was used to assess $CD4^+$ T cells for cell death (n = 6 per group). Data are presented as mean ± SEM. Student's t-test were used for statistical analysis. ns not significant.

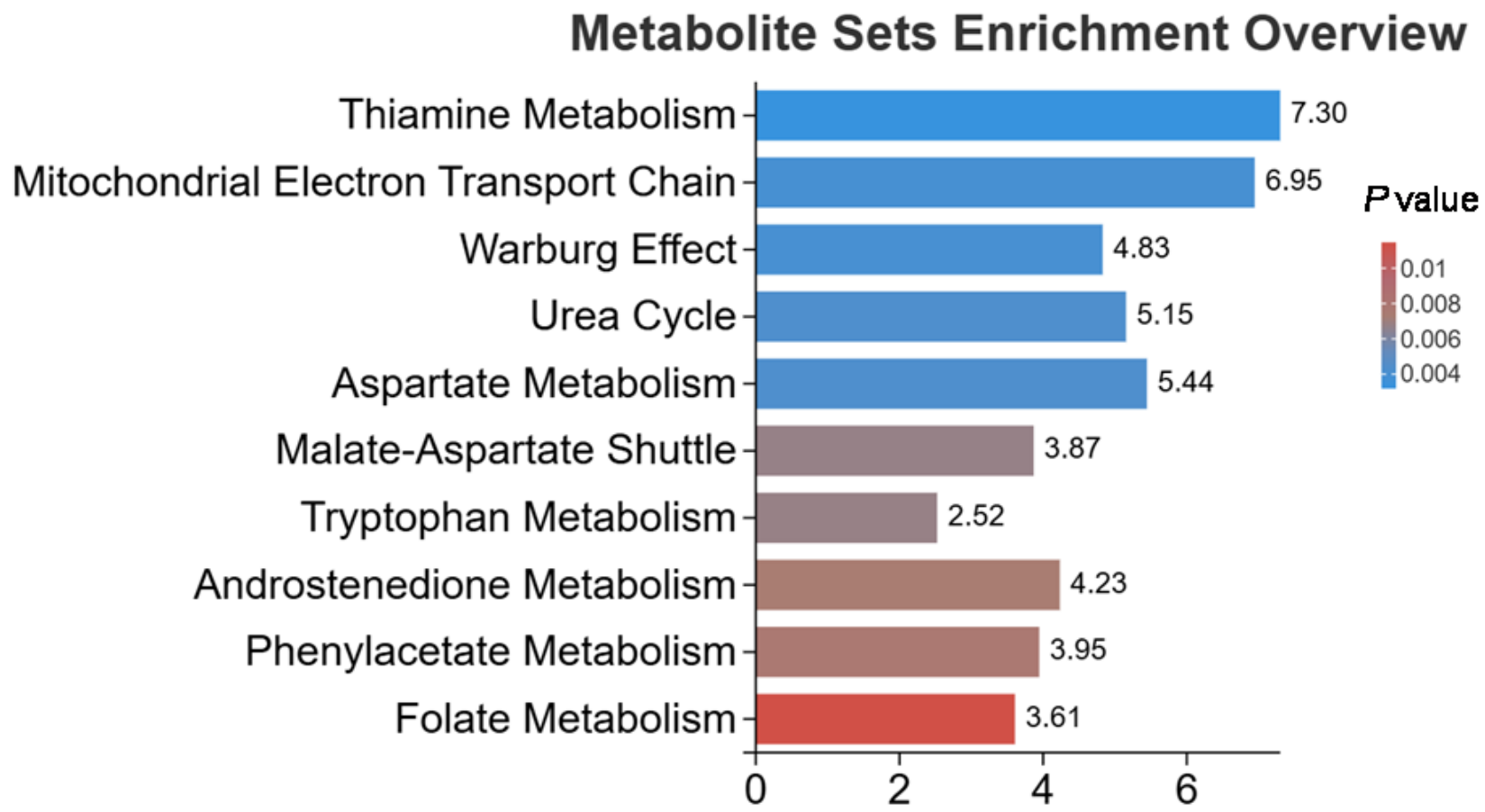

Figure S8. Metabolite sets enrichment analysis in ATAAD patients.
Serum samples from ATAAD patients were collected at the start of surgery and on postoperative day 1 for metabolomic analysis. The subsequent metabolite sets enrichment analysis revealed the pathways shown.

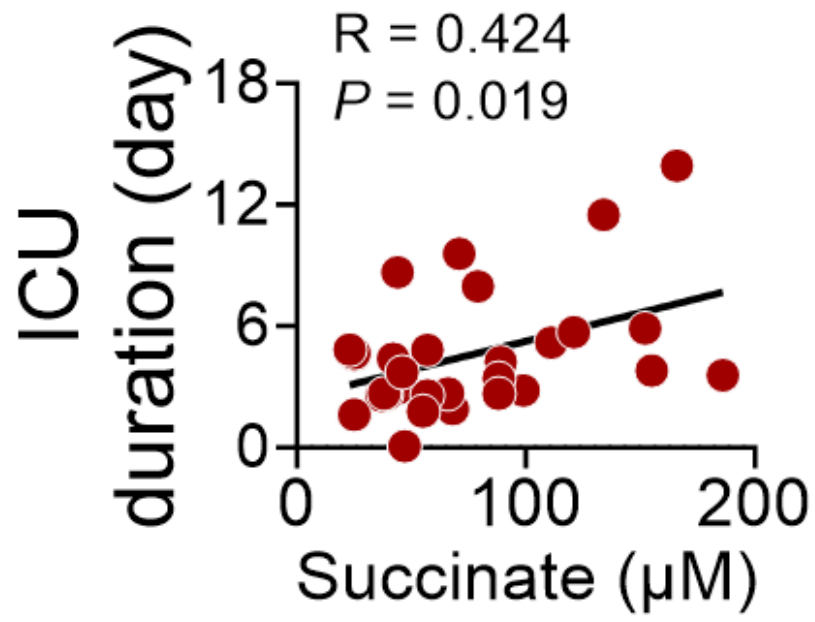

Figure S9. Correlation between POD1 serum succinate and ICU length of stay in ATAAD.

Correlation analysis of postoperative day 1 (POD1) serum succinate and ICU length of stay in ATAAD (n = 30). Data are presented as median (IQR) or mean ± SEM. Statistical analyses were performed using Pearson correlation.

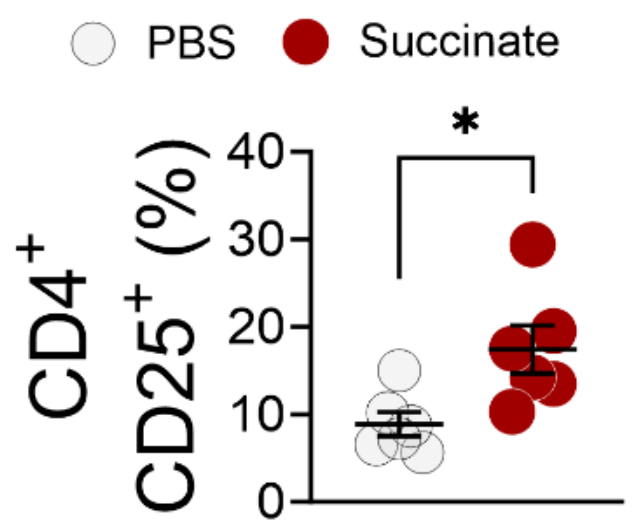

Figure S10. CD25 expression in $CD4^+$ T cells following succinate stimulation.
$CD4^+$ T cells were isolated from PBMCs of ATAAD patients and cultured under different conditions to evaluate activation by flow cytometry. Cells were cultured for 24 h with PBS or succinate (10 mM). Each point represents an individual patient. Data are shown as mean ± SEM. Statistical significance was determined using Student's t-test. $^*P < 0.05$.

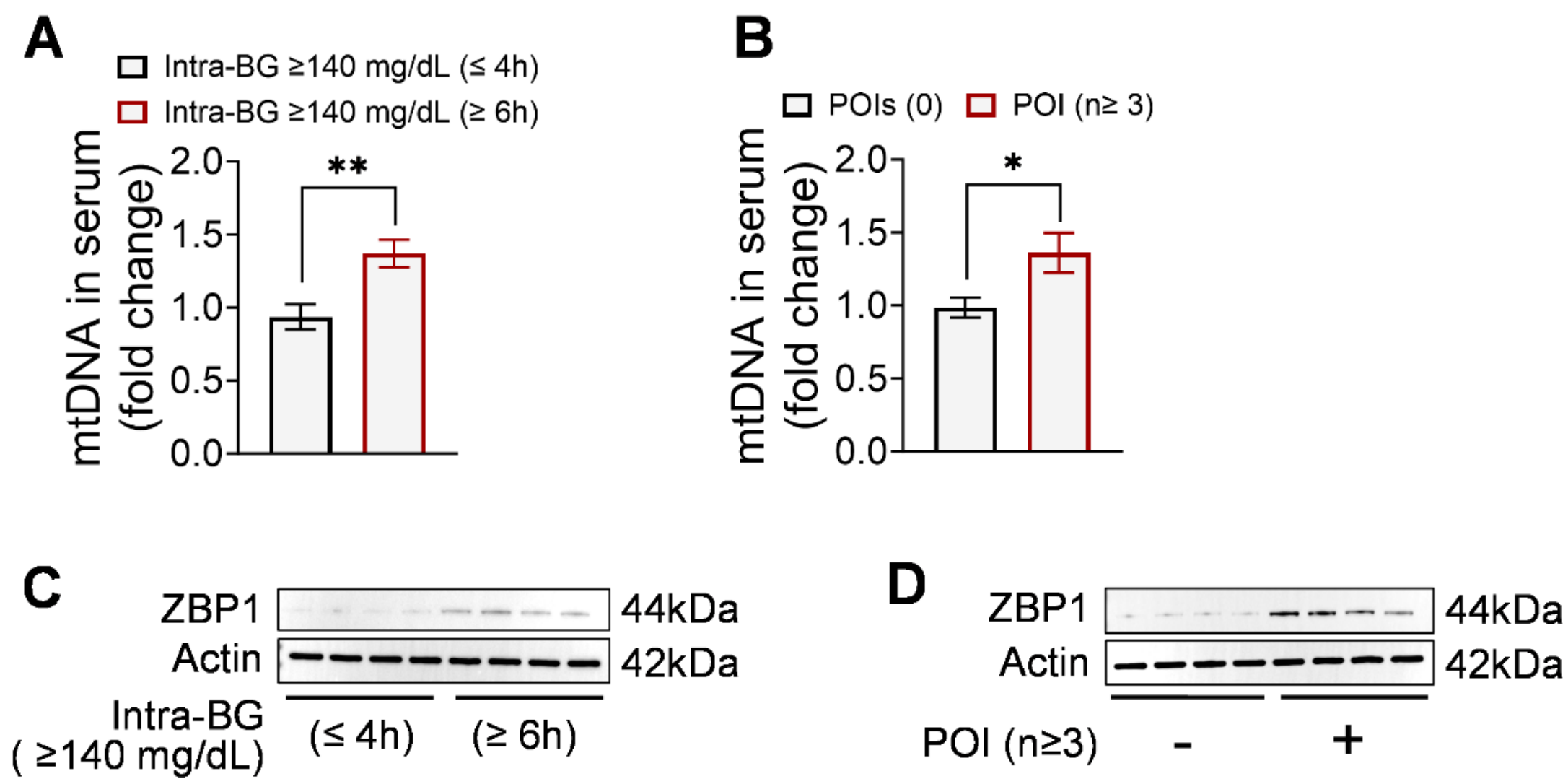

Figure S11. Association of serum mtDNA and ZBP1 expression in $CD4^+$ T cells with intraoperative hyperglycemia and POI in ATAAD patients.

A) Serum mtDNA levels, quantified by RT-qPCR, are compared across patients grouped by the duration of intraoperative hyperglycemia (≤ 4 h vs. ≥ 6 h n=6 per group). **B**) Serum mtDNA levels on postoperative day 1 (POD1) are compared across patient groups stratified by the number of postoperative organ injuries (POIs) (n=6 per group). **C, D**) Western blot analysis of ZBP1 in $CD4^+$ T cells isolated on POD1 across ATAAD patient groups stratified by the duration of intraoperative hyperglycemia (n=4 per group) or the number of POIs (n = 4 per group). Data are presented as mean ± SEM. Student's t-test were used for statistical analysis. ** $P < 0.01$.

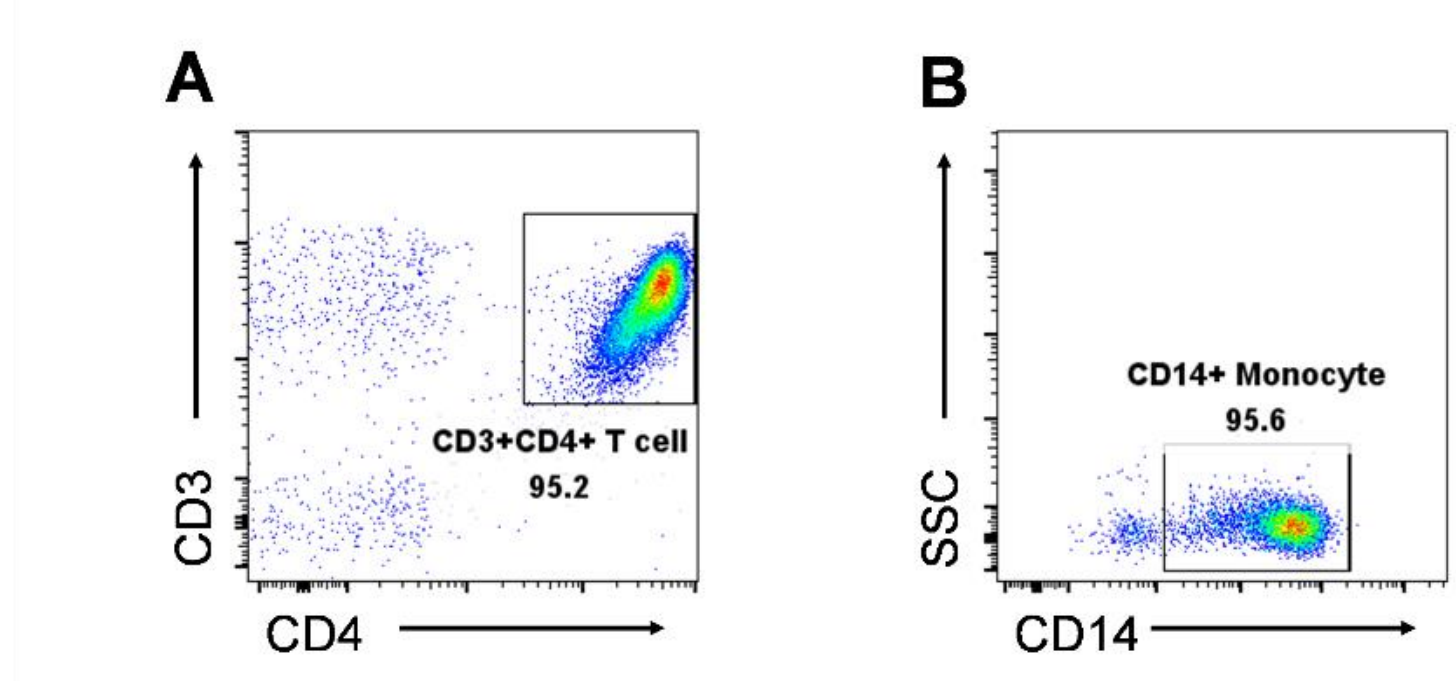

Figure S12. Flow cytometric assessment of the purity of isolated immune cell subsets.

A, B) $CD3^{+}CD4^{+}$ T cells (**A**), $CD14^{+}$ monocytes (**B**) were isolated using magnetic bead separation from ATAAD, with flow cytometry analysis indicating a purity greater than 95%.

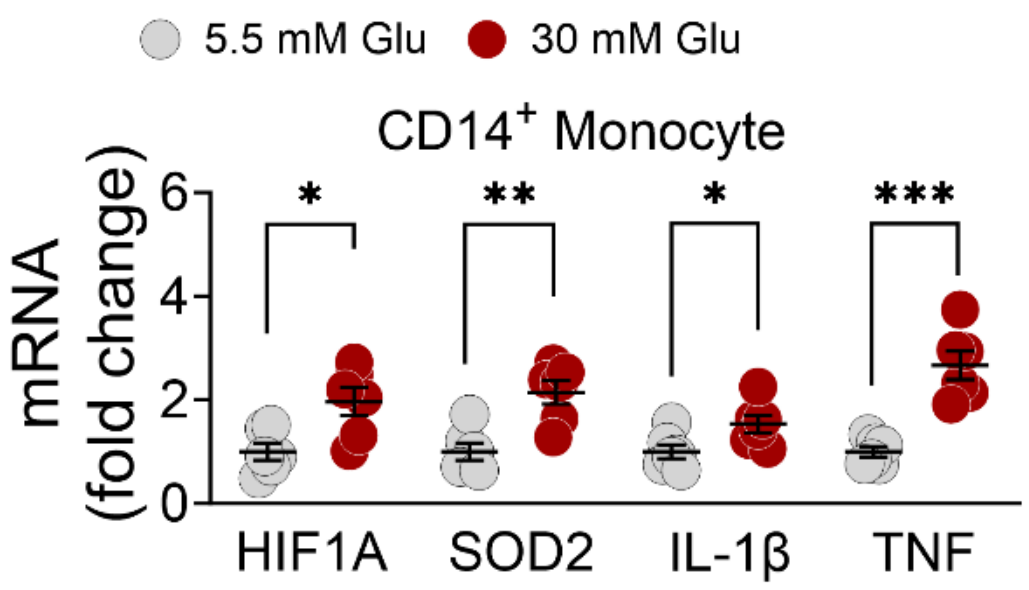

Figure S13. Pro-inflammatory and hypoxia-associated gene expression in human $CD14^+$ monocytes under high-glucose conditions.

$CD14^+$ monocytes were magnetically isolated from ATAAD patients and cultured for 24 h in 5.5 mM or 30 mM glucose. The mRNA levels of HIF1A, SOD2, IL-1β, TNF were measured by RT–qPCR (n = 6 per group). Data are presented as mean ± SEM. Statistical significance was determined using two-tailed Student's t-tests. $^*P < 0.05$; $^{**}P < 0.01$; $^{***}P < 0.001$.

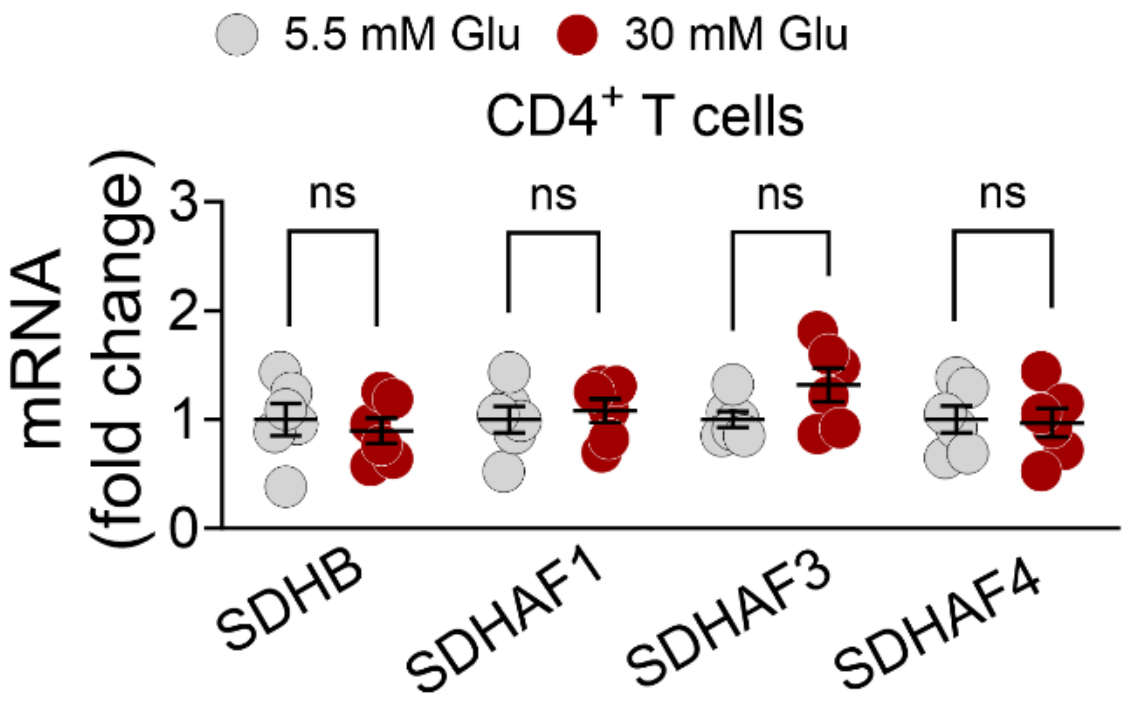

Figure S14. Gene expression of SDH subunits and assembly factors in CD4⁺ T cells under high-glucose conditions.

CD4⁺ T cells were magnetically isolated from ATAAD patients and cultured for 24 h in 5.5 mM or 30 mM glucose. The mRNA levels of SDHB, SDHAF1, SDHAF3, SDHAF4 were measured by RT–qPCR (n = 6 per group). Data are presented as mean ± SEM. Statistical significance was determined using two-tailed Student's t-tests. ns not significant.

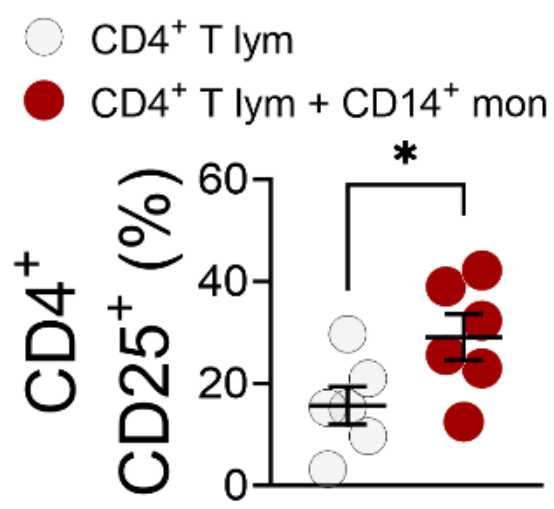

Figure S15. CD25 expression in CD4$^+$ T cells cultured with or without CD14$^+$ monocytes under high-glucose conditions.

CD4$^+$ T cells were isolated from PBMCs of ATAAD patients and cultured either alone or in co-culture with autologous CD14$^+$ monocytes under high-glucose conditions (30 mM glucose) for 24 h. CD4$^+$ T-cell activation was assessed by flow cytometric quantification of CD25$^+$ cells among CD4$^+$ T cells. Each point represents an individual patient. Data are shown as mean ± SEM. Statistical significance was determined using Student's t-test. *P < 0.05.

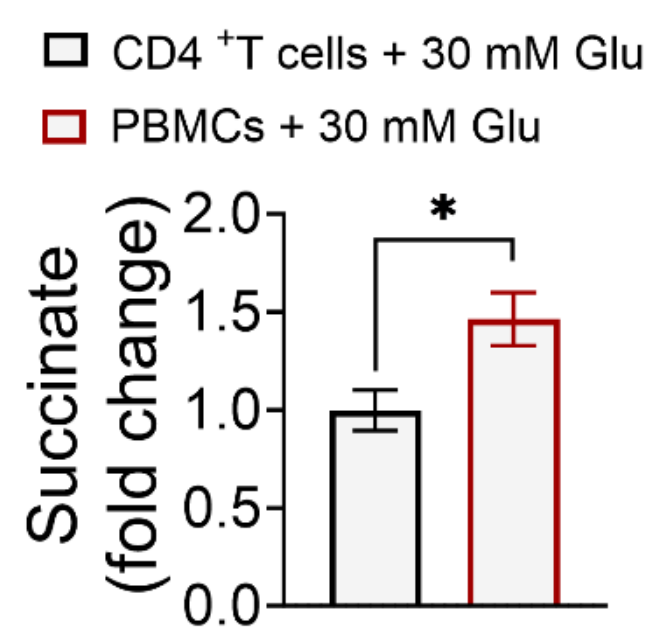

Figure S16. Intracellular succinate level in CD4⁺ T cells under high-glucose conditions.

CD4⁺ T cells were magnetically isolated from ATAAD patients and cultured for 24 h in 30 mM glucose. For the PBMC-priming group, PBMCs were cultured with the 30 mM glucose condition for 24 h, then isolating CD4⁺ T cells from PBMCs. Succinate levels in CD4⁺ T cells were measured using a colorimetric assay (n = 6 per group). Data are presented as mean ± SEM. Statistical significance was determined using two-tailed Student's t-tests. $^*P < 0.05$.

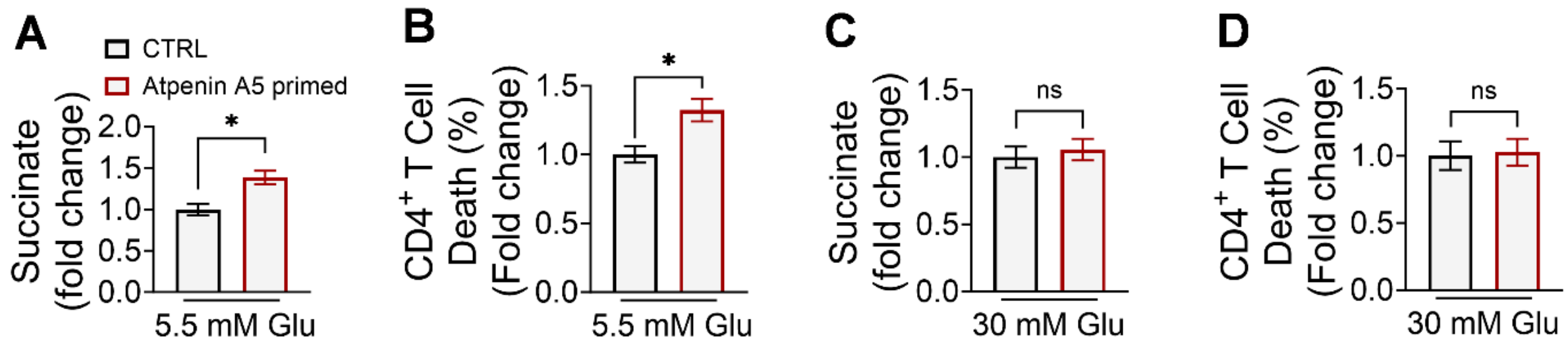

Figure S17. The effect of Atpenin A5-primed $CD14^+$ monocytes on $CD4^+$ T lymphocytes in a high-glucose environment.

A-D) $CD14^+$ monocytes and $CD4^+$ T cells were isolated from ATAAD patients. Monocytes were primed with Atpenin A5 (SDH inhibitor) for 24 h, then co-cultured with $CD4^+$ T cells for an additional 24 h in either 5.5 mM glucose or 30 mM glucose. Intracellular succinate in sorted $CD4^+$ T cells from co-cultures was measured and reported as relative change (**A, C**; n = 6 per group). Flow cytometry was used to assess $CD4^+$ T-cell death (**B, D**; n = 6 per group). Data are presented as mean ± SEM. Statistical significance was determined using two-tailed Student's t-tests. ns not significant. $^*P < 0.05$.

Table S1. Demographic Characteristics of ATAAD Patients with and without POIs

	**POIs**	**Non-POIs**	***P*-value**
No. (%) of patients	201 (54.3)	169 (45.7)	
Age, mean ± SD, years	50.87 ± 10.43	48.8 ± 10.97	0.107
Male, n (%)	149 (72.6)	118 (69.8)	0.566
Medical history, n (%)			
Smoking	86 (42.8)	57 (33.7)	0.293
Hypertension	150 (74.6)	113 (66.9)	0.108
Marfan syndrome	5 (2.6)	1 (0.8)	0.225
Coronary heart disease	24 (11.94)	12 (7.10)	0.158
Cerebral vascular disease	15 (7.5)	7 (4.1)	0.194
Symptom onset to hospital presentation, median (IQR), h	54.31 (24.3-61.7)	51.19 (34.3-74.7)	0.274
Preop creatinine (IQR), μmol/L	99 (73-132.1)	80 (65.80-104.4)	0.001
Preop transaminase (IQR), U/L	22.30 (14.75-34.85)	20.6 (13.95-40.90)	0.665
Preoperative CRP (mg/mL)	60.90 (11.20-123.0)	44.85 (12.18-127.5)	0.570
Operation time (h)	8.42 (7.00-10.0)	7.67 (5.58-9.04)	0.002
CPB (h)	3.98 (3.00-4.86)	3.67 (2.25-4.46)	0.016
Peak intraop lactate (mmol/L)	6.60 (5.40-8.30)	6.70 (5.63-8.18)	0.457
Transfusion (ml)	300 (0.00-600.0)	200 (0.00-505.00)	0.164
ICU stay (day)	4.95 (3.59-8.64)	2.89 (1.97-4.17)	< 0.001
Ventilation time (day)	2.22 (0.91-3.71)	1.68 (0.90-2.92)	< 0.001
Intraop BG			
Mean, median (IQR), mg/dL	165.6 (143.5-186.8)	155.7 (136.6-173.7)	0.008
Peak, median (IQR), mg/dL	226.8 (198.0-259.2)	213.3 (183.6-251.1)	0.062
BG>140mg/dL, median (IQR), h	3.98 (3.00-4.87)	3.47 (2.08-4.27)	< 0.001
Postop BG			
Mean, median (IQR), mg/dL	184.0 (159.6-217.4)	169.7 (147.6-193.9)	< 0.001
Peak, median (IQR), mg/dL	235.8 (207.5-275.4)	230.4 (191.7-279.0)	0.385
Preop immune counts median (IQR)			
White Blood Cell, 10^9/L	12.47 (10.65-14.91)	11.92 (10.19-13.93)	0.103
Neutrophils, 10^9/L	10.95 (8.92-13.06)	10.35 (8.34-11.80)	0.034
Monocyte, 10^9/L	0.69 (0.48-0.93)	0.63 (0.44-0.93)	0.289
Lymphocyte, 10^9/L	0.91 (0.69-1.13)	1.070 (0.85-1.33)	< 0.001
Postop immune counts (within 24 h), median (IQR)			
White Blood Cell, 10^9/L	11.24 (9.44-13.51)	11.41 (9.98-13.68)	0.516
Neutrophils, 10^9/L	10.11 (8.46-12.13)	10.34 (8.82-12.38)	0.362
Monocyte, 10^9/L	0.61 (0.435-0.835)	0.54 (0.38-0.73)	0.050
Lymphocyte, 10^9/L	0.36 (0.24-0.55)	0.52 (0.32-0.67)	0.002

Total transfusion volume was calculated as the sum of intraoperative platelet, plasma, and packed red blood cell transfusion volumes. Data are presented as number (%), mean ± SD or median (IQR). Depending on the types of data, the Student's *t*-test or Mann-

Whitney test or $\chi 2$ test or Fisher's exact test was applied, and $P < 0.05$ was considered to indicate statistical significance.

Table S2. Univariate Analysis of ATAAD Patients with and without POIs

Risk Factor	OR Value and 95% CI	*P*-value
CPB (h)	1.183 (1.022-1.376)	0.026
Preop creatinine, mg/dL	1.435 (1.043-2.096)	0.041
Operation time (h)	1.092 (0.999-1.196)	0.055
Transfusion (ml)	1.000 (0.999-1.001)	0.166
Intraop Mean BG, mg/dL	1.015 (1.013-1.318)	0.034
Intraop BG > 140, mg/dL (h)	1.226 (1.057-1.431)	0.008
Postop Mean BG, mg/dL	1.011 (1.005-1.018)	0.001
Preop Lymphocyte, 10^9/L	0.633 (0.359-1.100)	0.108
Preop Neutrophils, 10^9/L	1.158 (0.981-1.144)	0.152
Postop Lymphocyte(day1), 10^9/L	0.385 (0.183-0.791)	0.010

Variables associated with the outcome at a significance level of $p < 0.2$ were advanced to a multivariate regression model. Statistical significance in the final model was defined as a two-sided $p < 0.05$. The results are reported as Odds Ratios (OR) with 95% Confidence Intervals (CI).

Table S3. Demographic Characteristics of ATAAD Patients with and without Lymphopenia in POD1

	Lymphopenia	Non-Lymphopenia	*P*-value
No. of patients, n (%)	315 (85.1)	55 (14.9)	
Age, mean ± SD, years	51.34 ± 11	45.5 ± 6.71	0.064
Male, n (%)	229 (72.6)	38 (69.1)	0.625
Medical history, n (%)			
Smoking	125 (39.7)	18 (32.7)	0.369
Hypertension	226 (71.7)	37 (67.3)	0.521
Marfan syndrome	5 (1.6)	1 (1.8)	> 0.999
Coronary heart disease	32 (10.1)	4 (7.3)	0.628
Cerebral vascular disease	20 (6.2)	2 (3.6)	0.755
Preop creatinine (IQR), μmol/L	82.40 (74.4-114.94)	76.00 (67.9-89.5)	0.245
Preop transaminase (IQR), U/L	21.45 (15.65-32.75)	18.6 (15.55-37.9)	0.463
Symptom onset to hospital presentation, median (IQR), h	55.81 (21.3-67.7)	50.19 (31.3-78.7)	0.163
Preoperative CRP (mg/mL)	71.8 (12.95-128.5)	28.1 (9.58-93.20)	0.0242
Operation time (h)	8.322 (6.67-10.01)	7.670 (6.04-9.01)	0.045
CPB (h)	3.92 (2.57-4.85)	3.46 (2.69-3.90)	0.017
Peak intraop lactate (mmol/L)	6.800 (5.50-8.40)	6.1 (5.35-6.90)	0.0953
Transfusion (ml)	350 (0.00-650)	275 (0.00-600)	0.3852
ICU stay (day)	6.36 (2.90-6.88)	4.83 (1.75-7.29)	0.039
Ventilation time (day)	1.835 (0.94-3.09)	1.28 (0.67-2.65)	0.005
Intraop BG			
Mean, median (IQR), mg/dL	169.7 (145.2-183.4)	152.3 (132-169.4)	0.056
Peak, median (IQR), mg/dL	231.8 (191.2-264.2)	219.3 (181-262.5)	0.097
BG>140mg/dL, median (IQR), h	4.141 (3.13-4.53)	3.463 (2.13-4.42)	< 0.001
Postop BG			
Mean, median (IQR), mg/dL	178.8 (155.4-210.5)	167.7 (147.0-185.1)	0.003
Peak, median (IQR), mg/dL	235.8 (207.5-275.4)	230.4 (191.7-279)	0.453
Preop immune counts median (IQR)			
White Blood Cell, 10^9/L	12.12 (11.13-15.13)	11.46 (10.1-13.31)	0.251
Neutrophils, 10^9/L	10.45 (8.24-12.63)	9.12 (8.47-10.14)	0.224
Monocyte, 10^9/L	0.69 (0.48-0.93)	0.63 (0.44-0.93)	0.163
Lymphocyte, 10^9/L	0.91 (0.70-1.19)	1.348 (0.95-1.64)	< 0.001
Postop immune counts (within 24 h), median (IQR)			
White Blood Cell, 10^9/L	11.24 (8.12-14.56)	10.12 (8.83-12.56)	0.414
Neutrophils, 10^9/L	10.11 (8.46-12.13)	9.34 (7.54-11.46)	0.352
Monocyte, 10^9/L	0.53 (0.36-0.85)	0.48 (0.36-0.68)	0.131
Lymphocyte, 10^9/L	0.34 (0.24-0.51)	0.87 (0.83-0.98)	< 0.001

Total transfusion volume was calculated as the sum of intraoperative platelet, plasma, and packed red blood cell transfusion volumes. Data are presented as number (%), mean ± SD or median (IQR). Depending on the types of data, the Student's *t*-test or Mann-Whitney test or $\chi 2$ test or Fisher's exact test was applied, and $P < 0.05$ was considered to indicate statistical significance.

Table S4. Univariate Analysis of ATAAD Patients with and without Lymphopenia in POD1

Risk Factor	OR Value and 95% CI	*P*-value
CPB (h)	1.179 (0.971-1.453)	0.109
Operation time (h)	1.172 (1.031-1.341)	0.018
Preoperative CRP (mg/mL)	1.007 (1.000-1.016)	0.059
Peak intraop lactate (mmol/L)	1.236 (0.982-1.616)	0.095
Intraop Max BG, mg/dL	1.094 (0.979-1.232)	0.126
Intraop Mean BG, mg/dL	1.214 (1.008-1.475)	0.046
Intraop BG $>$ 140, mg/dL (h)	1.374 (1.135-1.700)	0.002
Postop Mean BG, mg/dL	1.468 (1.185-1.830)	0.001
Preop Lymphocyte, 10^9/L	0.326 (0.151-0.687)	0.003

Variables associated with the outcome at a significance level of $p < 0.2$ were advanced to a multivariate regression model. Statistical significance in the final model was defined as a two-sided $p < 0.05$. The results are reported as Odds Ratios (OR) with 95% Confidence Intervals (CI).

Table S5. Reagents

Reagents	Catalog	Manufacturer
CD4 MicroBeads, human	130-045-101	Miltenyi Biotec
CD14 MicroBeads, human	130-097-052	Miltenyi Biotec
Human $CD4^+$ T Cell Isolation Kit	19662	STEMCELL
Human Monocyte Isolation Kit	19669	STEMCELL
RPMI Medium 1640	8122128	Gibco
L-Glutamine	A2916801	Gibco
Penicillin-Streptomycin	15140122	ThermoFisher
PBS	10010023	Gibco
Mannitol	HY-N0378	MedChemExpress
D-Glucose	HY-B0389	MedChemExpress
Lymphoprep™	07851	Stemcell
N-acetylcysteine (NAC)	S1623	Selleck
Necrostatin-1 (NEC)	S8037	Selleck
Z-VAD-FMK	S7023	Selleck
Diethyl succinate	S6209	Selleck
MCC950	S7809	Selleck
VX765	S2228	Selleck
PHA	R30852801	InvivoGen
Lymphoprep	07851	Stemcell
ROS Detection Kit	88-5930-74	ThermoFisher
Δψm assay Kit JC-10	421902	Biolegend
ELISA human IL-1β	DY201	R&D Systems
ELISA human IL-6	D6050	R&D Systems
ELISA human TNF-α	DTA00D	R&D Systems
Trizol	15596026	ThermoFisher
Reverted Aid First Strand cDNA Synthesis Kit	K1622	ThermoFisher
SYBR Green	K0253	Thermo Fisher
Absolute Human Mitochondrial DNA Copy Number Quantification qPCR Assay Kit	8948	Sciencell
Succinate Assay Kit	MAK335	Sigma-Aldrich
FITC Annexin V Apoptosis Detection Kit with 7-AAD	640922	Biolegend
ATP Assay Kit	ab83355	Abcam
mitochondrial isolation kit	89874	Thermo Fisher
mtDNA isolation kit	ab65321	Abcam
Lipofectamine 2000	11668019	Thermo Fisher
IP Lysis Buffer	87788	Thermo Fisher
protein A/G magnetic beads	B23201	Selleck

Table S6. Antibody

Antibody	Catalog	Manufacture
APC/Cyanine7 anti-human CD45 Antibody	368515	Biolegend
PE/Cyanine7 anti-human CD3 Antibody	300419	Biolegend
PerCP/Cyanine5.5 anti-human CD4 Antibody	357413	Biolegend
APC anti-human CD4 Antibody	980812	Biolegend
FITC anti-human CD69 Antibody	310903	Biolegend
APC anti-human CD25 Antibody	567316	BD Pharmingen
BV510 anti-human CD8 Antibody	563256	BD Pharmingen
BV421 anti-human CD14 Antibody	563743	BD Pharmingen
FITC-AnnexinV	556419	BD Pharmingen
PerCP/Cyanine5.5-7AAD	420404	Biolegend
Precision Count Beads	424902	Biolegend
caspase-3	9662	CST
cleaved caspase-3	9664	CST
caspase-8	4790	CST
cleaved caspase-8	85171-3-RR	Proteintech
caspase-1	84735-1-RR	Proteintech
cleaved caspase-1	4199	CST
GSDMD-N	ab215203	Abcam
GSDMD	ab219800	Abcam
MLKL	14993	CST,
pMLKL	91689	CST
RIPK3	13526	CST
pRIPK3	93654	CST
ZBP1	13285-1-AP	Proteintech
SDHB	10620-1-AP	Proteintech
RIPK3	sc-374639	Santa Cruz
NLRP3	15101	CST
ASC	10500-1-AP	Proteintech
ZBP1	60968	CST
β-actin	81115-1-RR	Proteintech
Anti-rabbit IgG, HRP-linked Antibody	7074	CST

Table S7. Staining combinations

Staining combinations
CD3-PE/Cyanine7；CD4-PerCP/Cyanine5.5; CD8-BV510
CD3-PE/Cyanine7；CD4-PerCP/Cyanine5.5; CD69-FITC; CD25-APC
CD3-PE/Cyanine7；CD4-APC; 7AAD-PerCP/Cyanine5.5; AnnexinV-FITC
CD3-PE/Cyanine7；CD4-PerCP/Cyanine5.5; FITC-DCF
CD3-PE/Cyanine7；CD4-PerCP/Cyanine5.5; FITC/PE-JC10

Table S8. Primer sequences for RT–qPCR analyses

Gene	Forward primer (5′-3′)	Reverse primer (5′-3′)
IL-1β	CTTTGAAGCTGATGGCCCTAAA	AGTGGTGGTCGGAGATTCGT
IL-6	GGCACTGGCAGAAAACAACC	GCAAGTCTCCTCATTGAATCC
TNF-α	GAGATCAATCGGCCCGACTA	CGTTTGGGAAGGTTGGATGT
18S	AGACAACAAGCTCCGTGAAGA	CAGAAGTGACGCAGCCCTCTA
HIF-1a	TCATCCAAGAAGCCCTAACGTG	TTTCGCTTTCTCTGAGCATTCTG
SOD2	TTTCAATAAGGAACGGGGACAC	GTGCTCCCACACATCAATCC
SDHB	GACCTTCTGGTAACTTCGGCT	TGGAGTCAATCATCCAGCGATAG
SDHAF1	CTTTGCCCTTTGGCCTTTGC	GATCGCGGTACAGGCTCAG
SDHAF3	TTGGTTCTGACGAGGCACAG	AGCTCCTGCAACTGTCCAAT
SDHAF4	AAGAAGCCGAAGTTACCAGAAG	CCCAATCTCCATATCGGGGAGG
