# Supplementary material for: Stress Hyperglycemia Drives CD4+ T Cell PANoptosis and Postoperative Organ Injury via Monocyte‐Derived Succinate
Source: Adv Sci (Weinh). 2026 Aug 5;13(60):e76968. doi: 10.1002/advs.76968 (PMC13440196; doi:10.1002/advs.76968)

Figure 2J

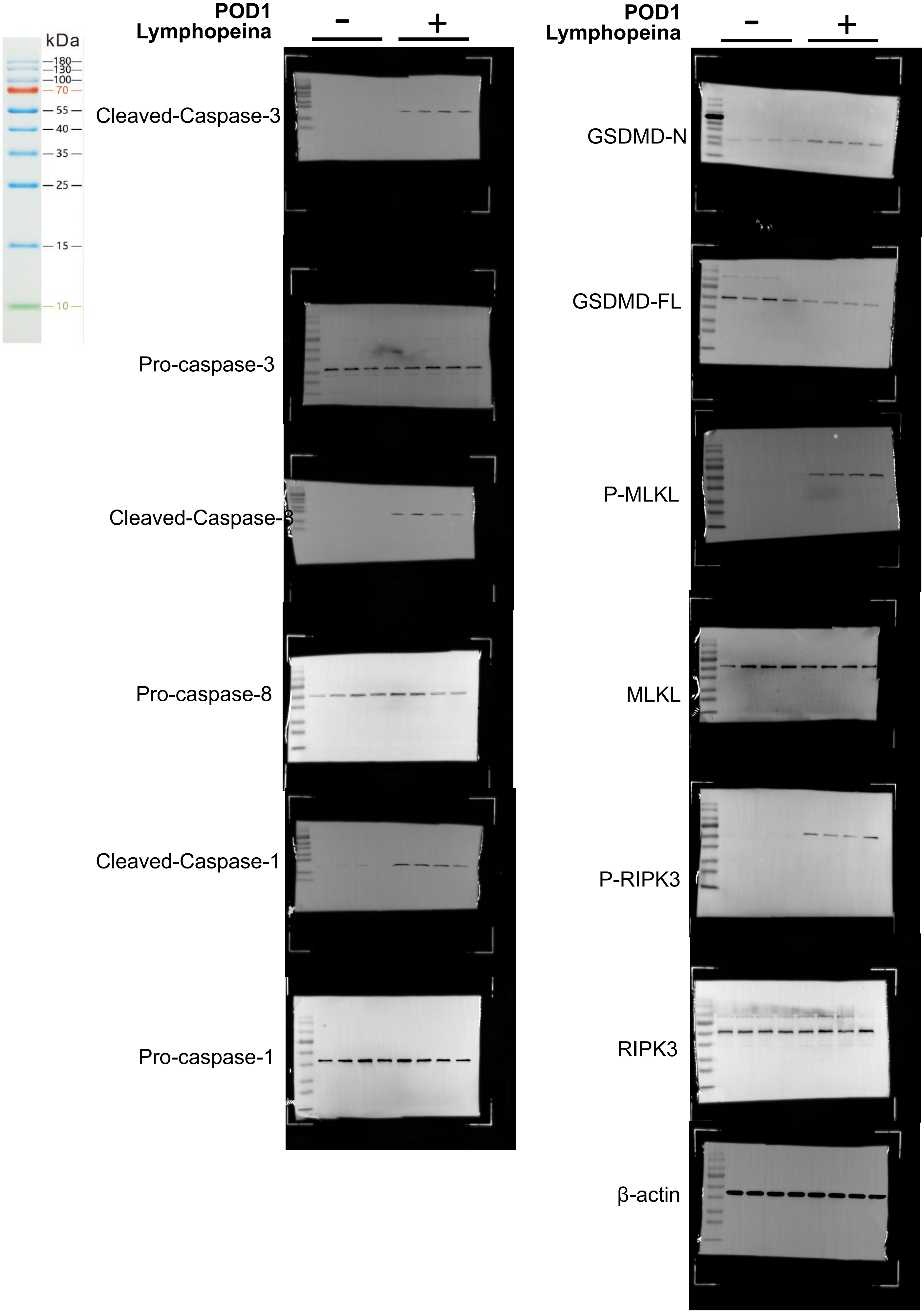
kDa
180
130
100
70
55
40
35
25
15
10
POD1
Lymphopeina
-
+
Cleaved-Caspase-3
Pro-caspase-3
Cleaved-Caspase-8
Pro-caspase-8
Cleaved-Caspase-1
Pro-caspase-1
POD1
Lymphopeina
-
+
GSDMD-N
GSDMD-FL
P-MLKL
MLKL
P-RIPK3
RIPK3
β-actin

Figure 2K

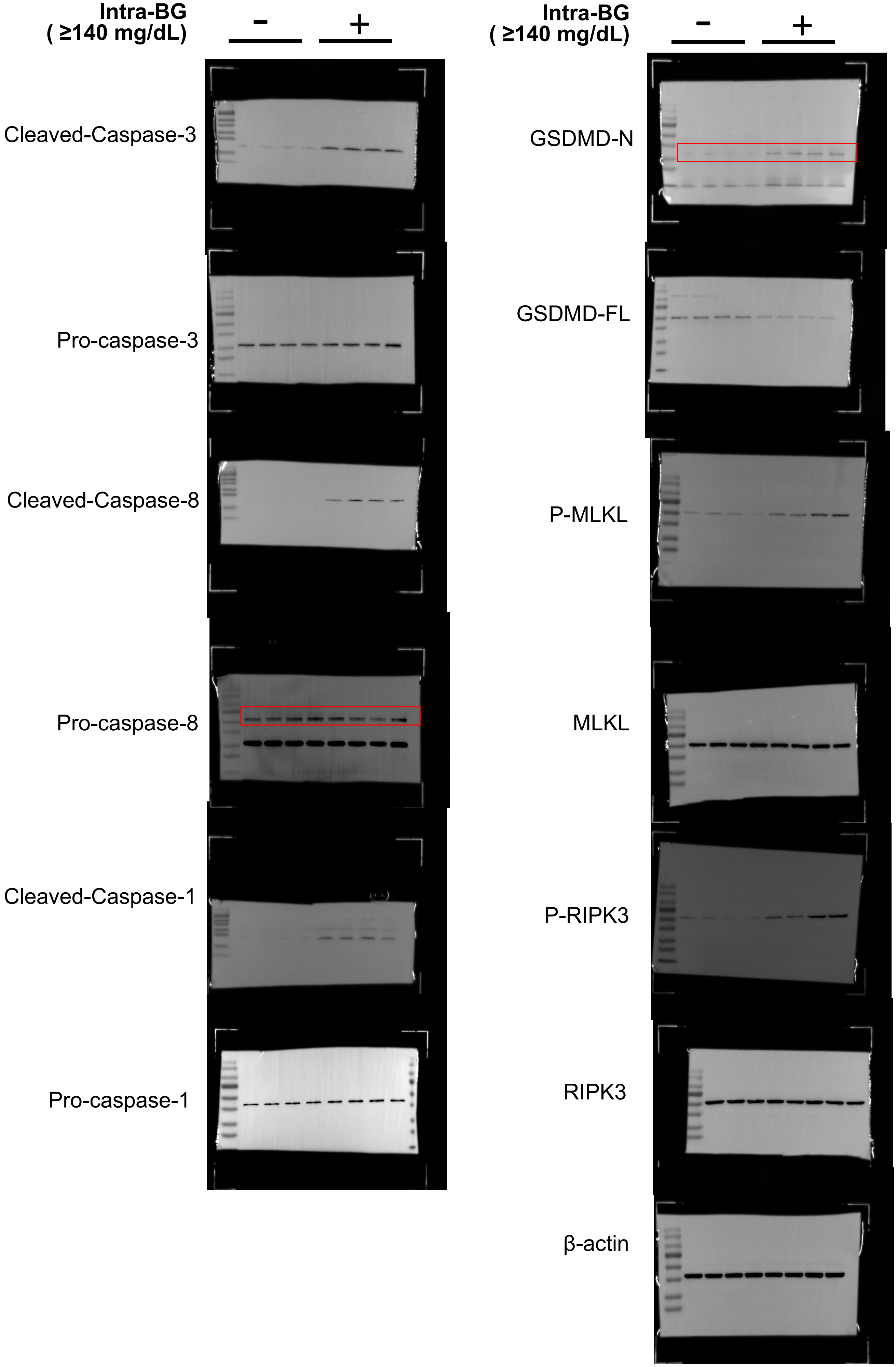
Intra-BG
(≥140 mg/dL)
-
+
Cleaved-Caspase-3
Pro-caspase-3
Cleaved-Caspase-8
Pro-caspase-8
Cleaved-Caspase-1
Pro-caspase-1
Intra-BG
(≥140 mg/dL)
-
+
GSDMD-N
GSDMD-FL
P-MLKL
MLKL
P-RIPK3
RIPK3
β-actin

Figure 3J

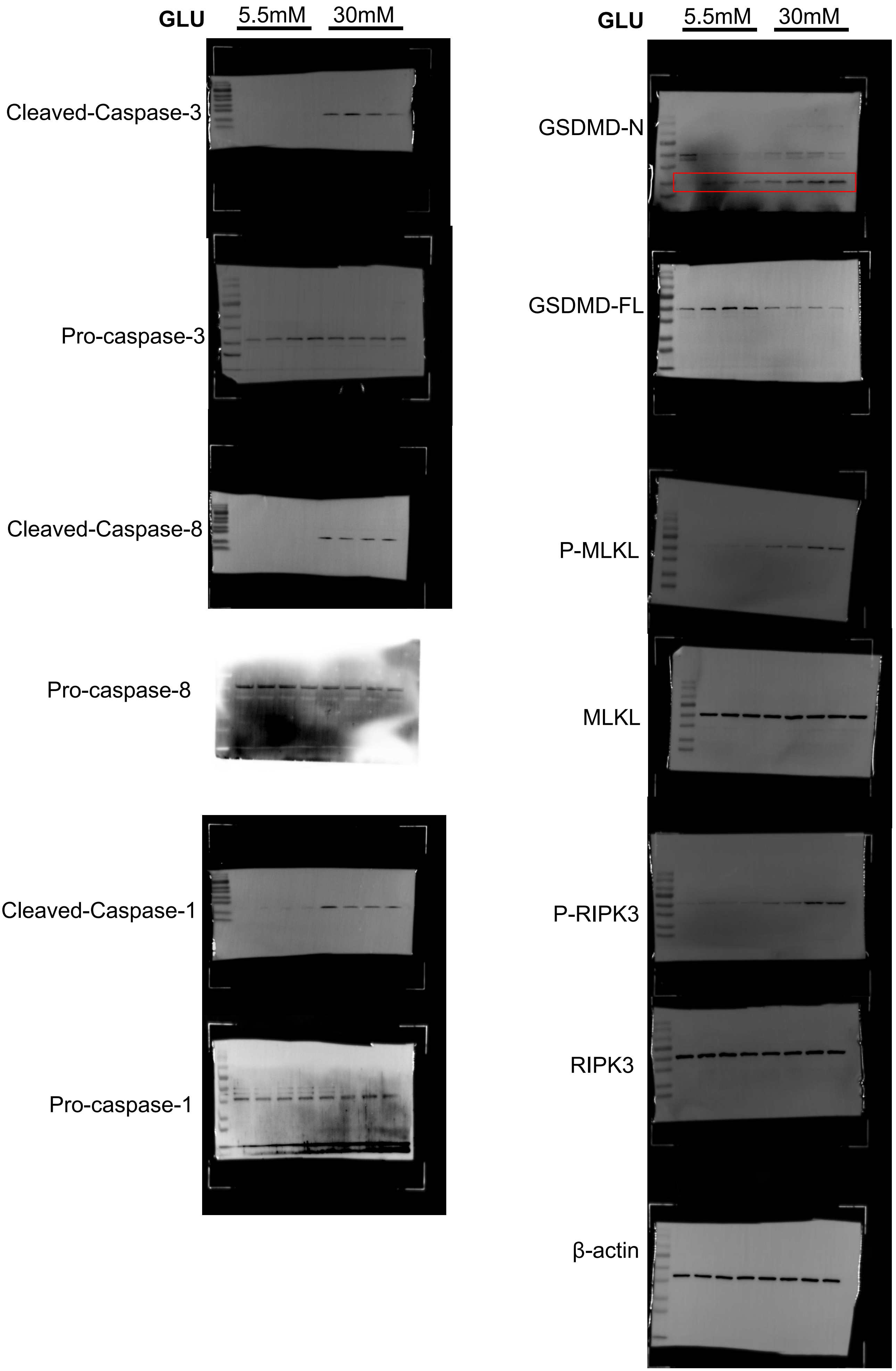
GLU
5.5mM
30mM
Cleaved-Caspase-3
Pro-caspase-3
Cleaved-Caspase-8
Pro-caspase-8
Cleaved-Caspase-1
Pro-caspase-1
GLU
5.5mM
30mM
GSDMD-N
GSDMD-FL
P-MLKL
MLKL
P-RIPK3
RIPK3
β-actin

Figure 4P

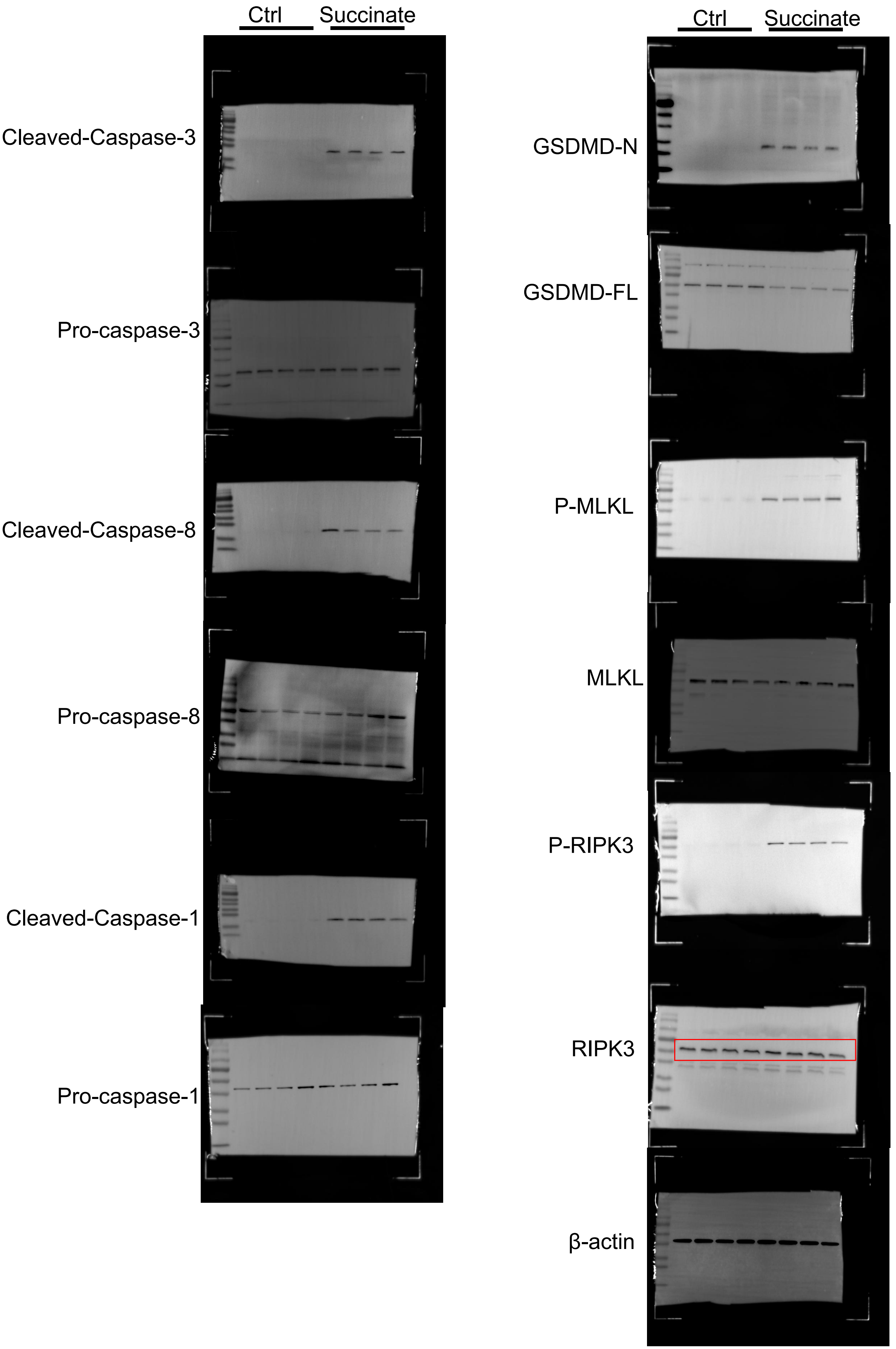

Ctrl
Succinate
Cleaved-Caspase-3
Pro-caspase-3
Cleaved-Caspase-8
Pro-caspase-8
Cleaved-Caspase-1
Pro-caspase-1
Ctrl
Succinate
GSDMD-N
GSDMD-FL
P-MLKL
MLKL
P-RIPK3
RIPK3
β-actin

Figure 5L

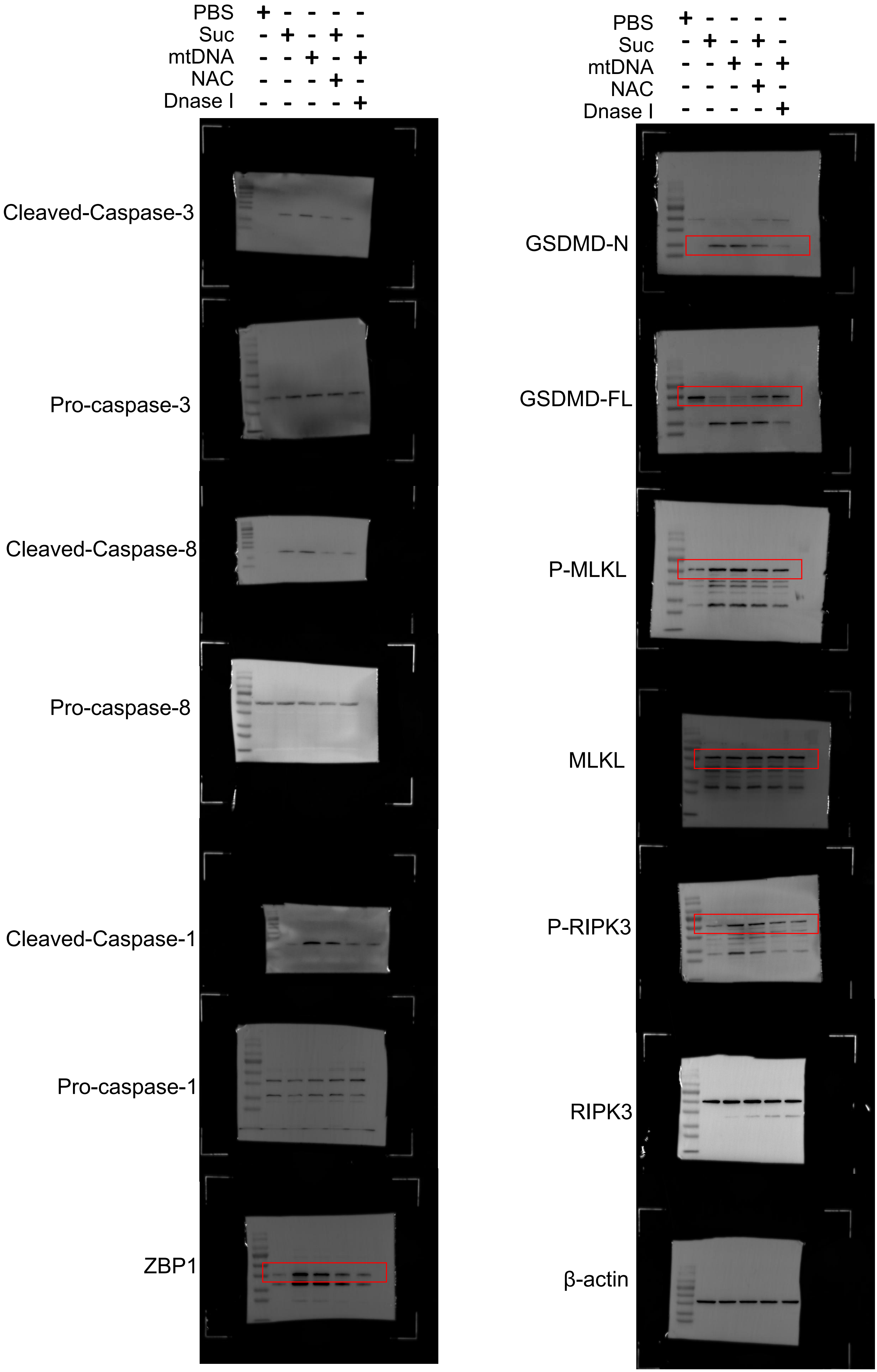

PBS + - - - -
Suc - + - + -
mtDNA - - + - +
NAC - - - + -
Dnase I - - - - +
Cleaved-Caspase-3
Pro-caspase-3
Cleaved-Caspase-8
Pro-caspase-8
Cleaved-Caspase-1
Pro-caspase-1
ZBP1
PBS + - - - -
Suc - + - + -
mtDNA - - + - +
NAC - - - + -
Dnase I - - - - +
GSDMD-N
GSDMD-FL
P-MLKL
MLKL
P-RIPK3
RIPK3
β-actin

Figure 5M

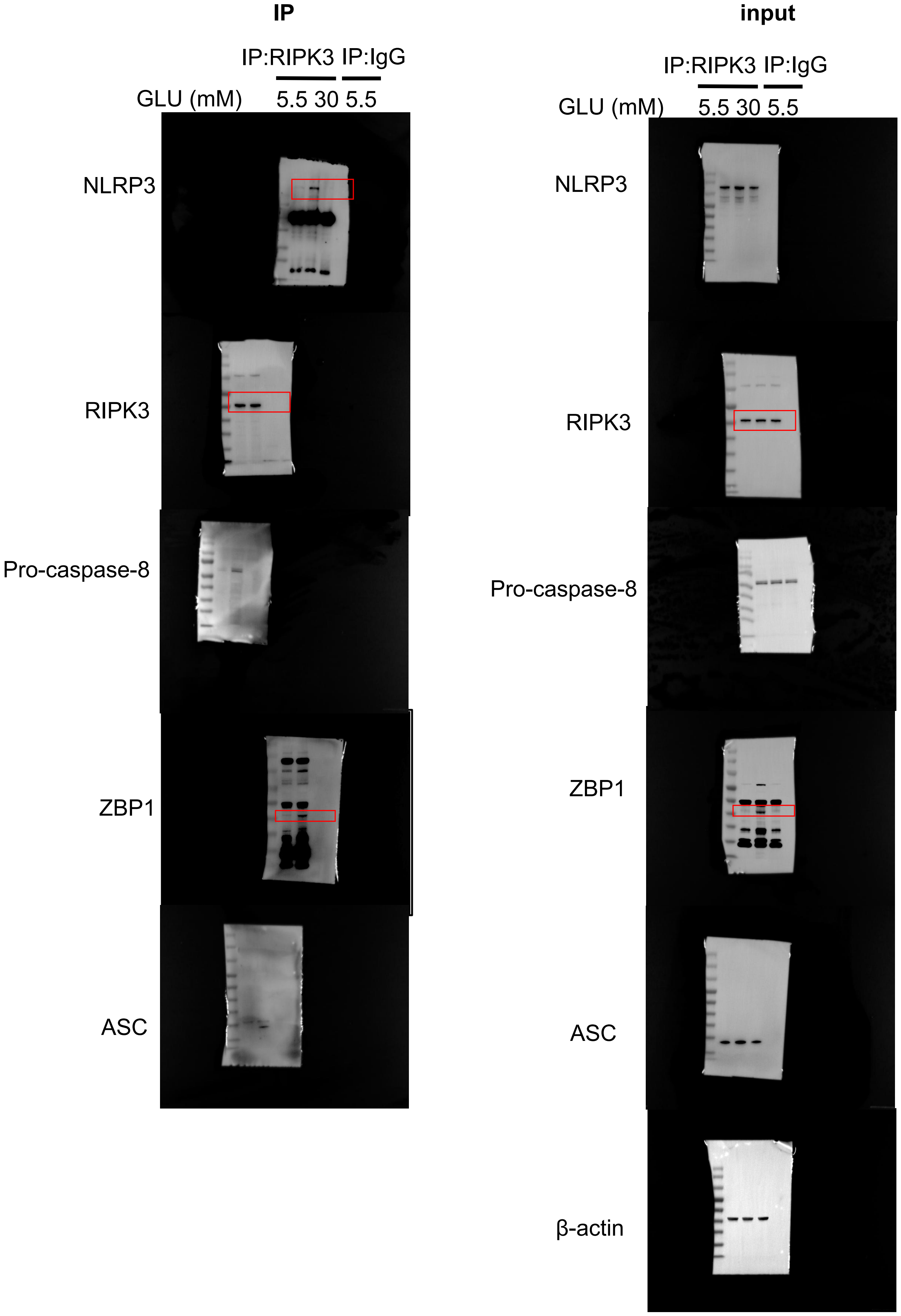
IP
IP:RIPK3 IP:IgG
GLU (mM) 5.5 30 5.5
NLRP3
RIPK3
Pro-caspase-8
ZBP1
ASC
input
IP:RIPK3 IP:IgG
GLU (mM) 5.5 30 5.5
NLRP3
RIPK3
Pro-caspase-8
ZBP1
ASC
β-actin

Figure 5N

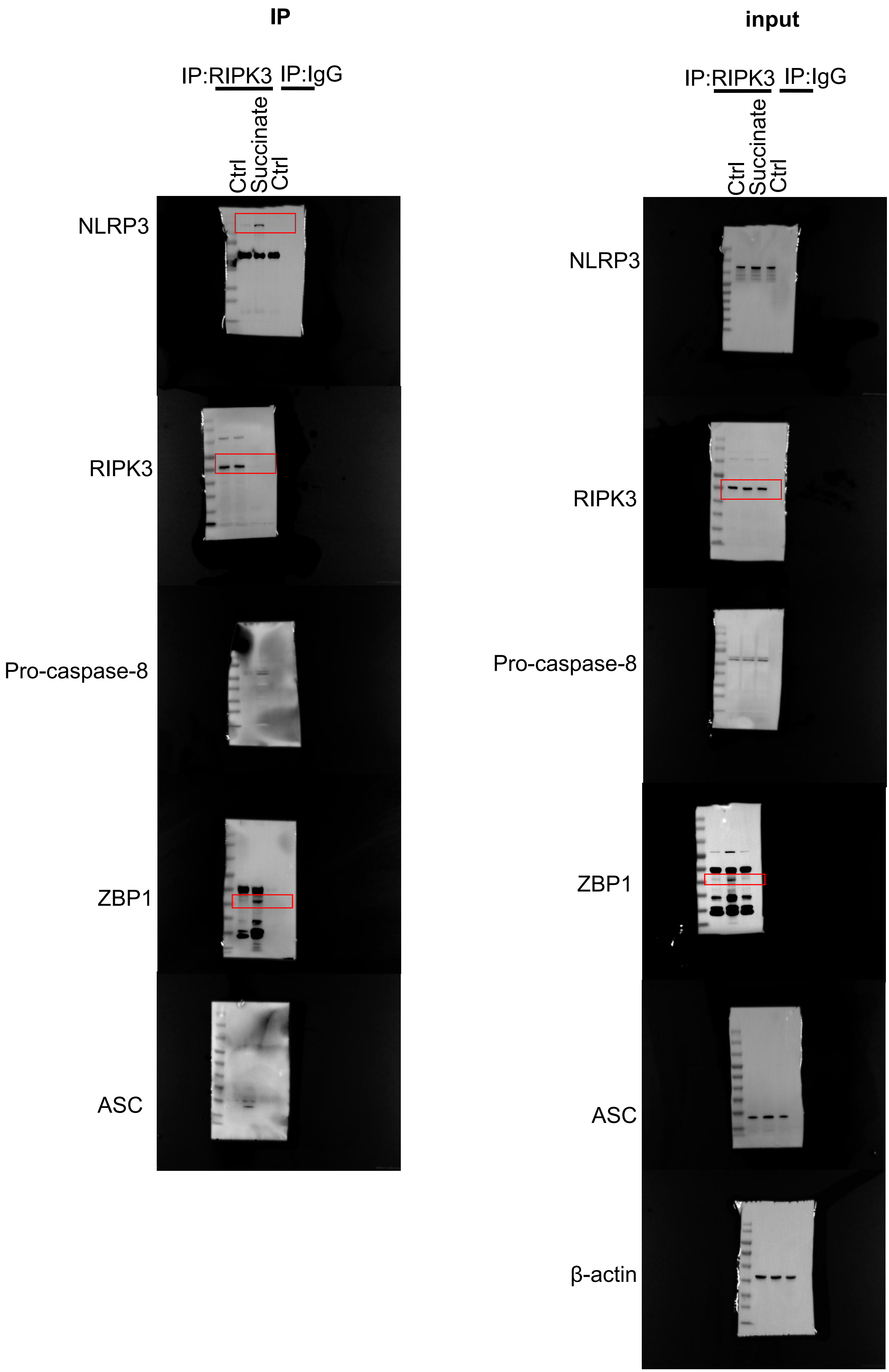
IP
input
IP:RIPK3
IP:IgG
Ctrl
Succinate
Ctrl
NLRP3
RIPK3
Pro-caspase-8
ZBP1
ASC
β-actin

Figure 5O

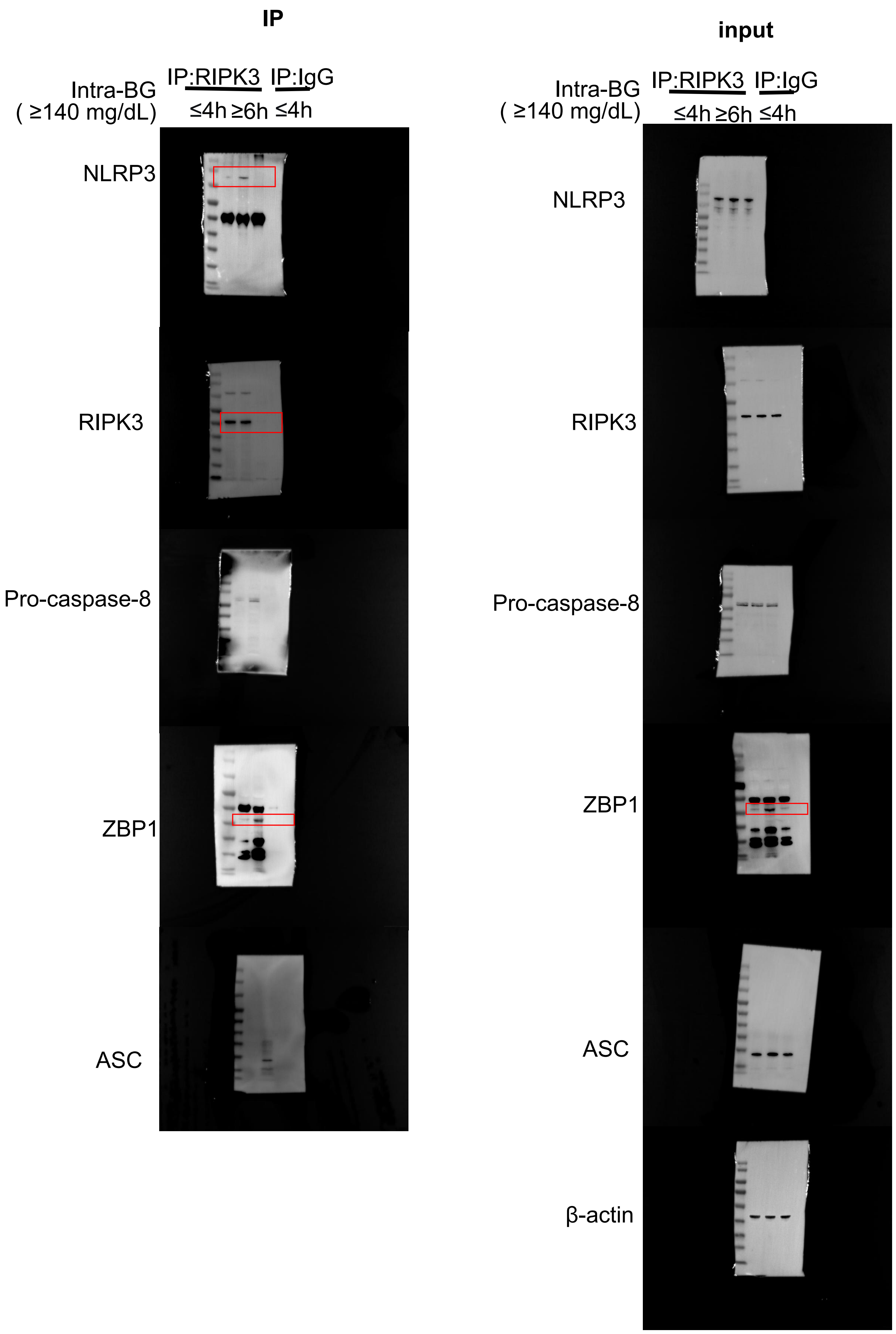
IP
input
Intra-BG
(≥140 mg/dL)
IP:RIPK3
IP:IgG
≤4h ≥6h ≤4h
NLRP3
RIPK3
Pro-caspase-8
ZBP1
ASC
β-actin

Figure 5P

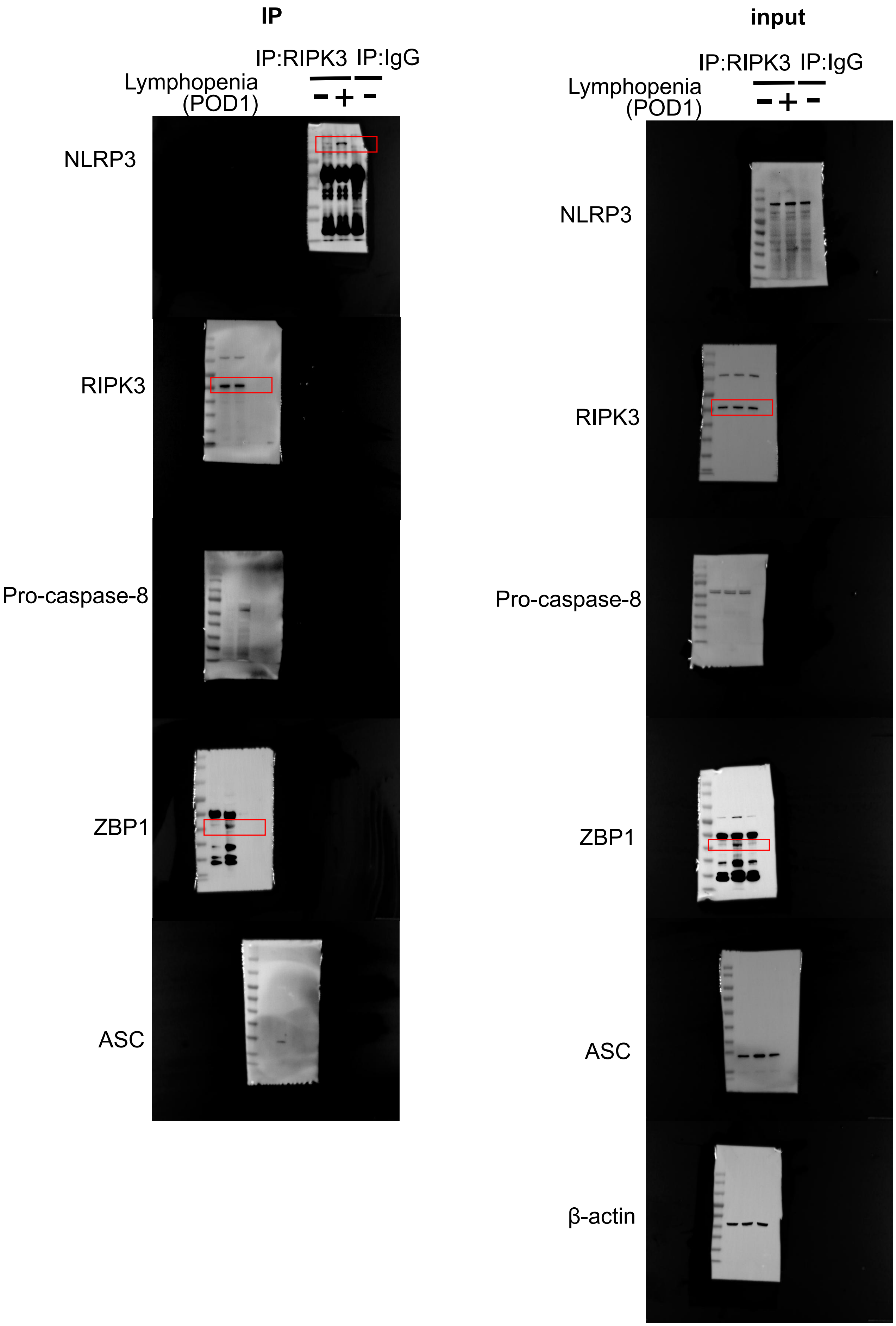
IP
IP:RIPK3 IP:IgG
Lymphopenia (POD1)
- + -
NLRP3
RIPK3
Pro-caspase-8
ZBP1
ASC
input
IP:RIPK3 IP:IgG
Lymphopenia (POD1)
- + -
NLRP3
RIPK3
Pro-caspase-8
ZBP1
ASC
β-actin

Figure 6G

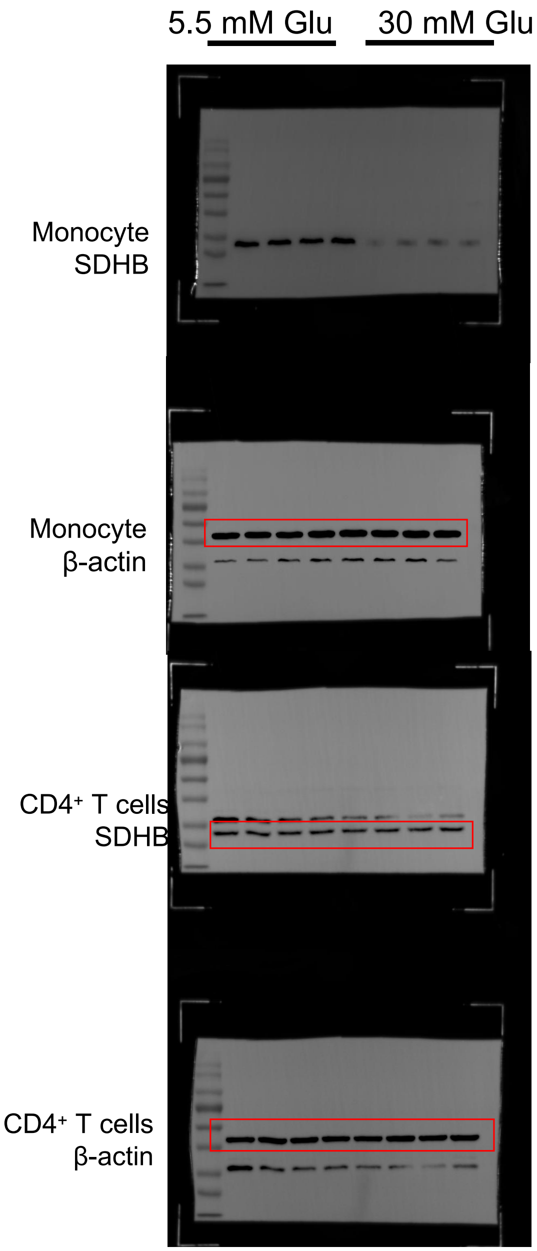

5.5 mM Glu
30 mM Glu
Monocyte SDHB
Monocyte β-actin
CD4+ T cells SDHB
CD4+ T cells β-actin

Figure 7K

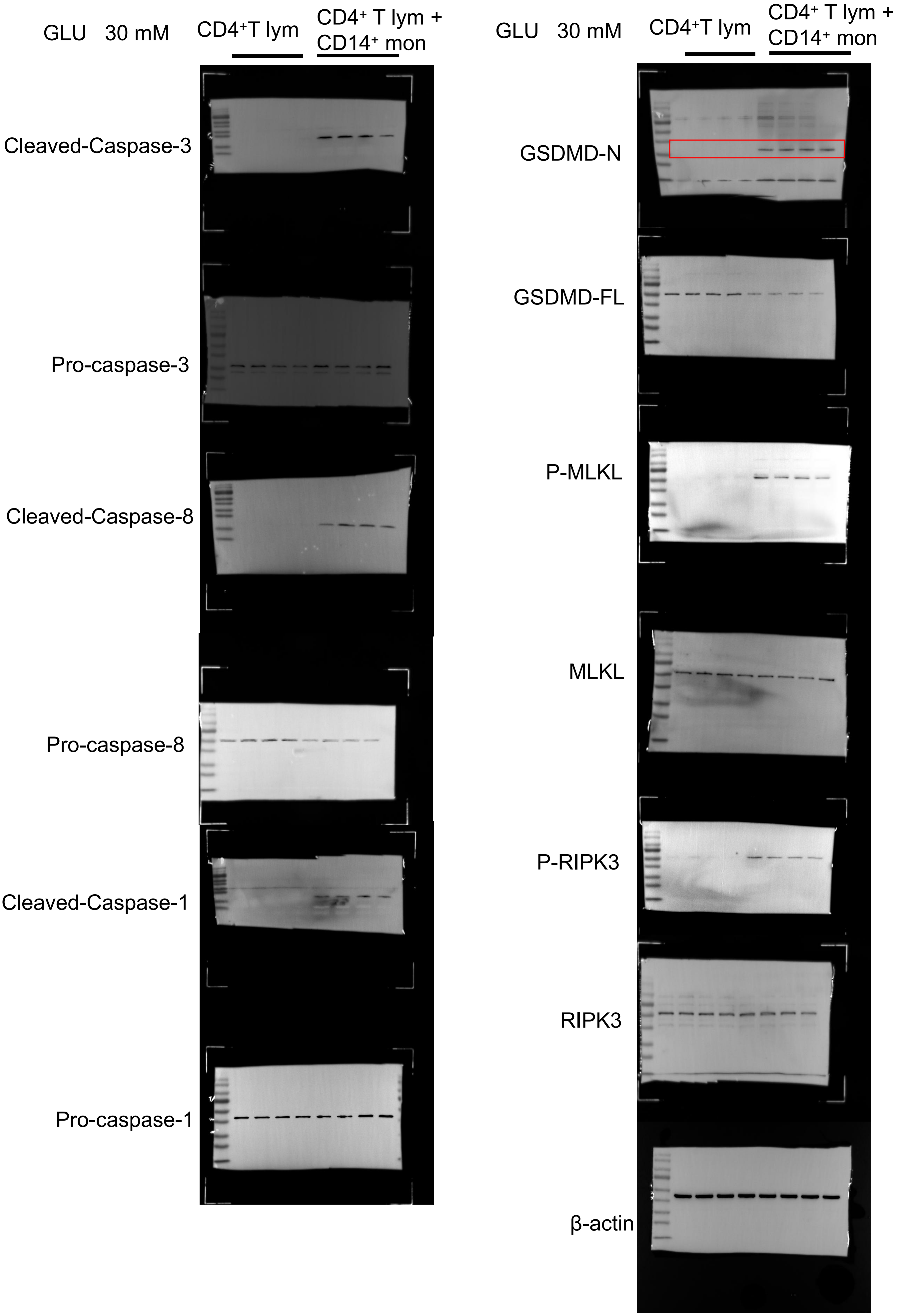

GLU 30 mM
CD4+T lym
CD4+ T lym + CD14+ mon
Cleaved-Caspase-3
Pro-caspase-3
Cleaved-Caspase-8
Pro-caspase-8
Cleaved-Caspase-1
Pro-caspase-1
GSDMD-N
GSDMD-FL
P-MLKL
MLKL
P-RIPK3
RIPK3
β-actin

Figure S11C

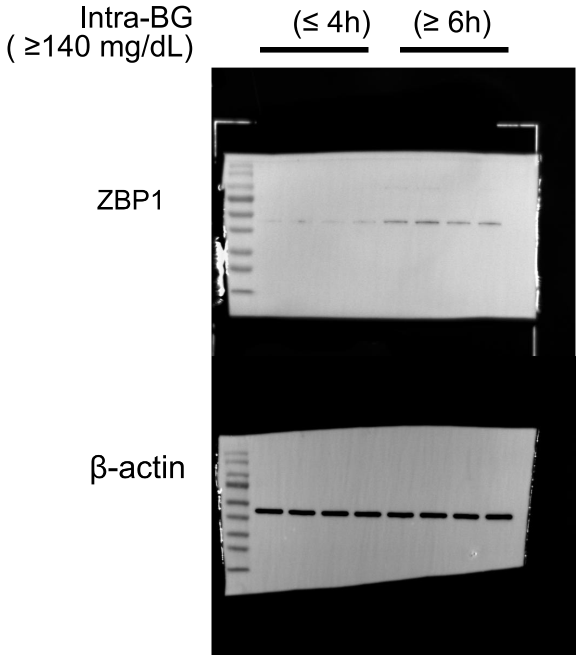

Figure S11D

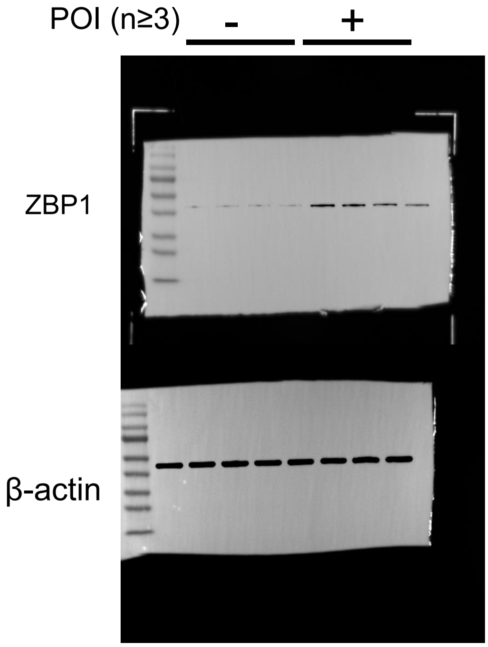

Supplement: Supplementary file 2 [file ADVS-13-e76968-s001.zip › Supplementary Materials 2. original Western blot.pdf]
